# Synthesis and Applications of Bicyclo[1.1.0]butyl and Azabicyclo[1.1.0]butyl Organometallics

**DOI:** 10.1002/chem.202300008

**Published:** 2023-04-14

**Authors:** Jasper L. Tyler, Varinder K. Aggarwal

**Affiliations:** ^1^ School of Chemistry University of Bristol Cantock's Close Bristol BS8 1TS UK

**Keywords:** azabicyclo[1.1.0]butane, bicyclo[1.1.0]butane, metalation, organometallics, strained molecules

## Abstract

The use of metalated (aza)bicyclo[1.1.0]butanes in synthesis is currently experiencing a renaissance, as evidenced by the numerous reports in the last 5 years that have relied on such intermediates to undergo unique transformations or generate novel fragments. Since their discovery, these species have been demonstrated to participate in a wide range of reactions with carbon and heteroatom electrophiles, as well as metal complexes, to facilitate the rapid diversification of (aza)bicyclo[1.1.0]butane‐containing compounds. Key to this is the relative acidity of the bridgehead C−H bonds which promotes facile deprotonation and subsequent functionalization of an unsubstituted position on the carbon framework via the intermediacy of a metalated (aza)bicyclo[1.1.0]butane. Additionally, the late‐stage incorporation of deuterium atoms in strained fragments has led to the elucidation of numerous reaction mechanisms that involve strained bicycles. The continued investigation into the inimitable reactivity of metalated bicycles will cement their importance within the field of organometallic chemistry.

## Graphical abstract

Metalated (aza)bicyclo[1.1.0]butanes are currently experiencing a renaissance, as evidenced by the numerous reports in the last 5 years that have relied on such intermediates to facilitate new reactivity or generate novel fragments. This review explores the broad range of chemical transformations that have utilized metalated (aza)bicyclo[1.1.0]butanes as linchpin reagents from their first reported use in synthesis to the present day.

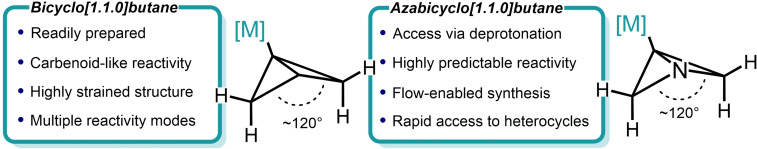

## 1 Introduction: Structure and Reactivity of (Aza)Bicyclo[1.1.0]butane

**Figure 1:**
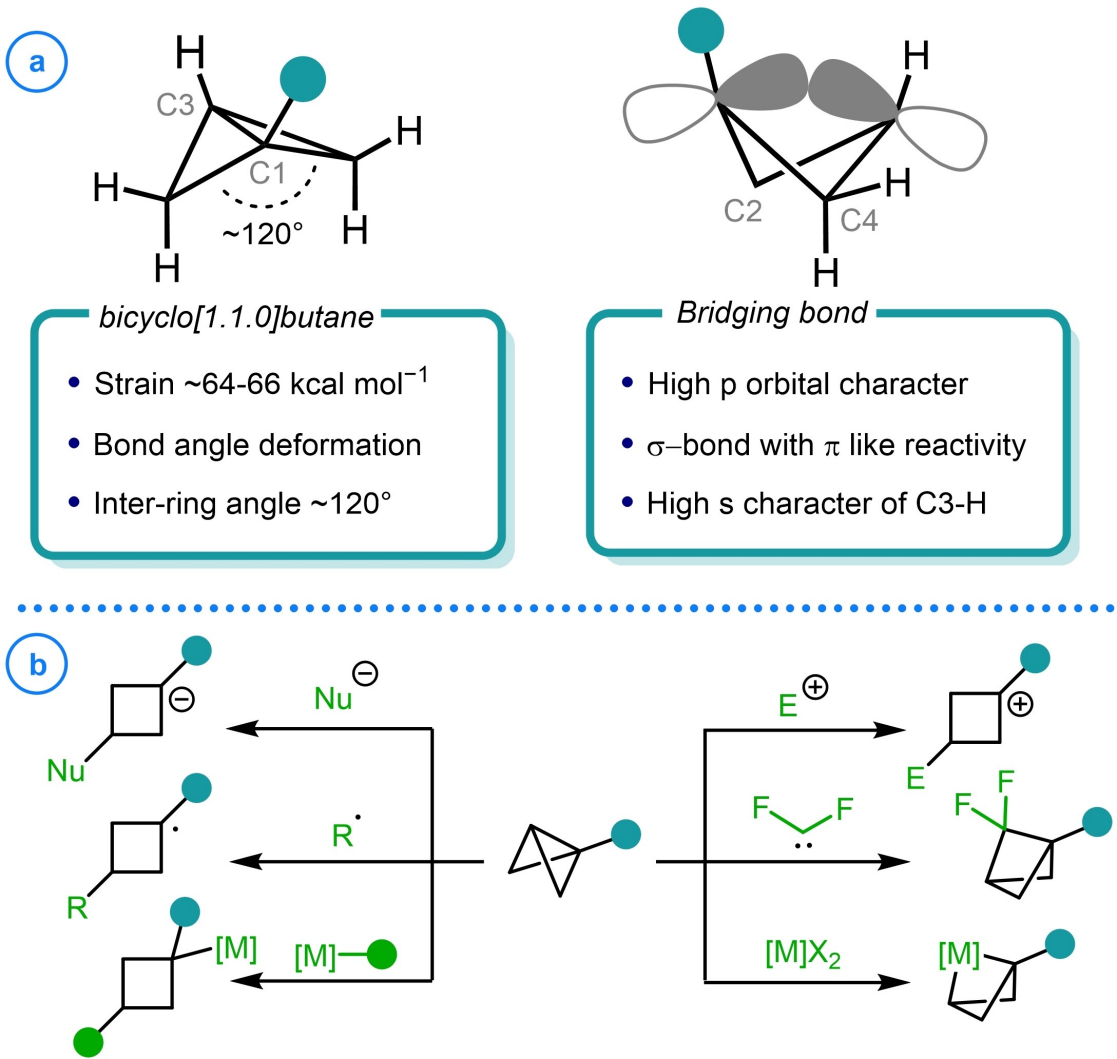
a) Structure and bonding of bicyclo[1.1.0]butane. b) Reactivity modes available to bicyclo[1.1.0]butane‐containing compounds.

Bicyclo[1.1.0]butane (BCB) represents the fewest atom containing example of a bridged compound, consisting of two fused three‐membered rings with an inter ring angle of approximately 120° (Figure 1a).[^1^, ^2^, ^3^] Despite their structural simplicity, the high strain energy these structures possess makes them valuable synthetic intermediates in the assembly of unique scaffolds.^[4]^ The strain energy exhibited by BCB has been quantified numerous times by both experimental and theoretical methods and typically falls in the range of 64‐66 kcal mol^−1^, exceeding the strain energy of the sum of two cyclopropane rings.[^1^, ^5^, ^6^, ^7^] The origin of this additional strain has been attributed to both 1,3‐carbon‐carbon non‐bonded repulsion due to the proximity of the C2 and C4 methylene units and to bond angle deformations at the bridgehead atoms.[^8^, ^9^] However, it has been shown that this strain energy is significantly perturbed by the presence of bridgehead substituents.^[10]^ As substitution at these positions is associated with little change to the bond angles of the structure, it has been stated that this is predominantly an electronic effect.^[11]^

The geometric restrictions imposed upon these bicyclic species means the bridgehead atoms adopt unusually tight bond angles.[^12^, ^13^] In order to satisfy the necessary geometry, the hybridization of the atoms gives rise to a central bond that is almost entirely comprised of p orbitals and bridgehead atom bonds that have increased s‐character.[^14^, ^15^] Analogous to the bonding observed with cyclopropane rings, these “bent” bonds place a significant proportion of electron density outside the internuclear axis.^[16]^ This model has been verified by theoretical and experimental studies that have demonstrated that the highly strained central σ‐bond can participate in conjugation. Consequently, the bridging bond of BCB exhibits π‐character^[18]^ which facilitates the reaction of these bicyclic species with nucleophiles,[^19^, ^20^, ^21^] electrophiles,[^1^, ^22^, ^23^] radicals,[^24^, ^25^, ^26^] metal complexes[^27^, ^28^, ^29^] and π‐systems in cycloaddition reactions (Figure 1b).[^30^, ^31^, ^32^]

On the other hand, the nitrogen‐containing analogue of BCB, 1‐azabicyclo[1.1.0]butane (ABB), has received much less attention, with the structural parameters often assumed to be equivalent to those of BCB.^[33]^ This is not an unreasonable prediction as x‐ray analysis has demonstrated the similarities between the bond angles and lengths of ABB and BCB which would suggest that the same hybridization models are valid for the aza‐analogue.[^34^, ^35^] Interestingly, recent work from Anderson and Duarte calculated the strain‐release energy (SRE) of the central bond of ABB to be −31.4 kcal mol^−1^, whereas the SRE for BCB was determined to be −40.2 kcal mol^−1^.^[36]^ In this report the authors state that strain‐release alone is an insufficient predictor of reactivity and a larger contribution to the kinetic barrier in strained systems comes from the extent of bond delocalization. Therefore, ABB and BCB should display a more similar level of reactivity than would be predicted based purely on strain‐release energy, as the level of electron delocalization, estimated by the number of three‐membered rings fused to the breaking bond, is comparable. However, the reduced thermodynamic driving force, as well as the presence of the nitrogen atom, is likely to significantly impact the activation barrier to reactivity.

In spite of their strained structure and strong thermodynamic driving force towards ring‐opening, bicyclo[1.1.0]butane‐containing compounds are often easily handled, readily purified and have even been isolated as products from natural enzymatic processes.^[37]^ The relative stability and plethora of reactivity modes available to this class of compound makes them highly valuable synthetic intermediates and have accordingly attracted much attention in recent years.[^4^, ^33^]

### 1.1 Metalated (aza)bicyclo[1.1.0]butanes

The assembly of (aza)bicyclo[1.1.0]butanes can be achieved by a variety of methods such nucleophilic substitution,[^1^, ^38^, ^39^, ^40^] cation‐induced ring‐closure,^[41]^ [2+1] cycloaddition reactions or through the use of sulfoxonium ylides,^[42]^ transition metals[^43^, ^44^] and enzyme catalysis.^[45]^ However, the construction of these neutral molecules lies outside the scope of the present treatise and has been comprehensively summarized in previous reviews.[^4^, ^33^] The specificity of the substrates and conditions required to construct such highly strained species can often limit the scope of any subsequent reactivity for these bicyclic molecules. In this regard, the metalation of (aza)bicyclo[1.1.0]butanes offers the unique potential to further elaborate highly strained fragments and access synthetic precursors that can more widely exploit the inimitable reactivity of the bridging bond. The abundance of scaffolds that can be accessed from these compact building blocks demonstrates the value of these intermediates, whose full potential is far from fully realized. In this review, the broad range of chemical transformations that have utilized metalated (aza)bicyclo[1.1.0]butanes as linchpin reagents will be discussed, from their first reported use in synthesis to the present day. Each discussion will focus on a different class of electrophilic coupling partner and how the resulting products have been harnessed to both improve existing syntheses and access novel structures.

### 1.2 Synthesis of metalated (aza)bicyclo[1.1.0]butanes

**Figure 2:**
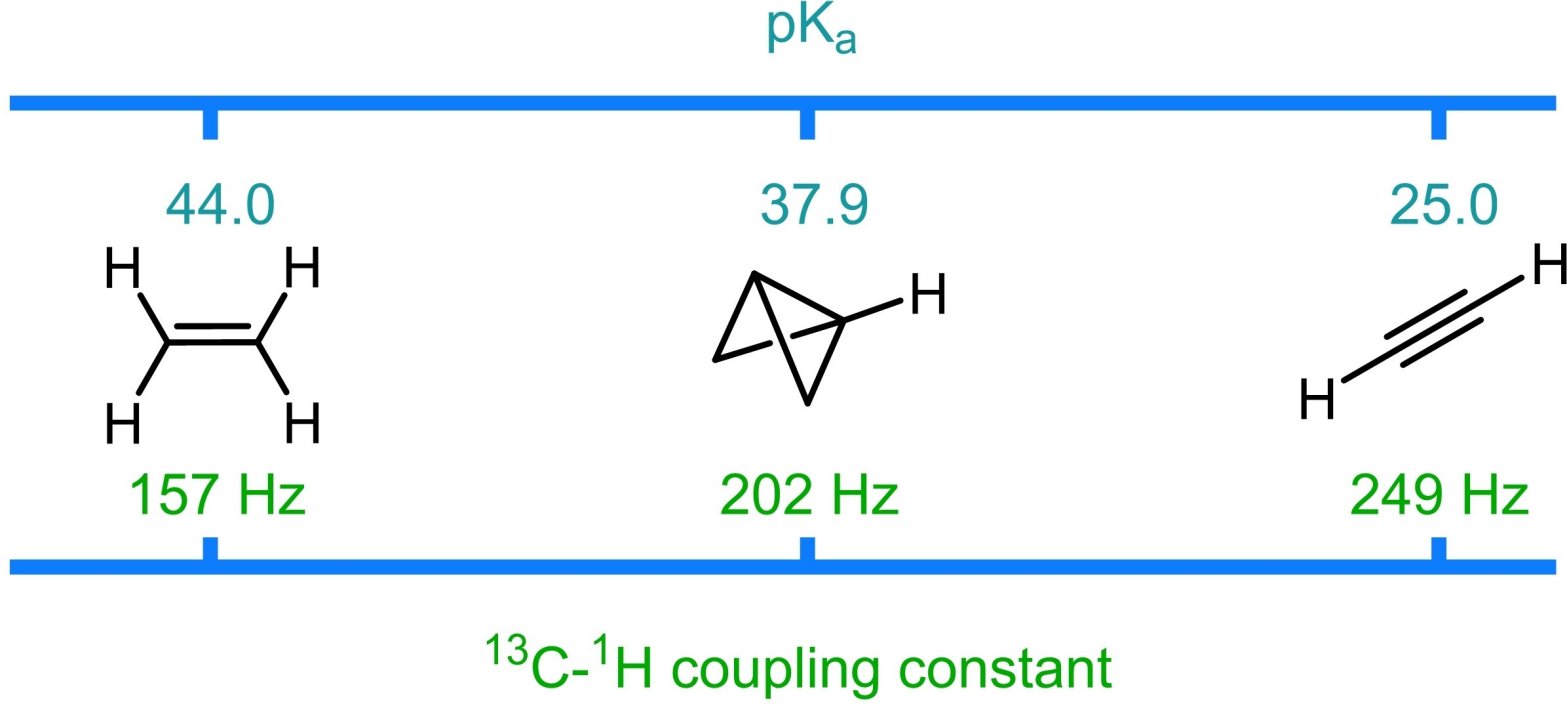
Calculated pK_a_ values and observed ^13^C−^1^H NMR coupling constants of ethylene, bicyclo[1.1.0]butane and ethyne.

As discussed above, the unusual bonding exhibited by bicyclo[1.1.0]butane translates into a unique hybridization mode for the atoms involved in the central bond.[^12^, ^13^] The enhanced s‐character of the bridgehead atoms would be predicted to result in an increase in the stabilization of negative charge at this position.[^14^, ^15^] Additionally, Dill and coworkers calculated that bridgehead lithiated BCB showed a significant reduction in strain energy (from 65.6 to 40.7 kcal mol^−1^) when compared to the unsubstituted parent compound.^[10]^ It was stated that this observation arises due to the σ‐donating ability of lithium which stabilises the σ‐framework of the strained bicycle. These effects correlate with the relative acidity of the bridgehead C−H bond in BCB which was calculated computationally by Alkorta and Elguero to have a pK_a_ of 37.9 (Figure 2).^[46]^ This value is considerably lower than for an unstrained sp^3^ hybridized carbon atom and lies in between the pK_a_ values for ethylene (44.0) and ethyne (25.0).^[46]^

**Figure 3:**
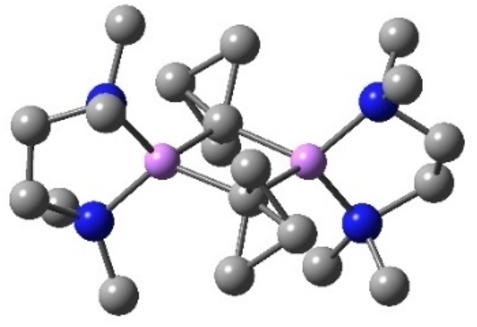
Dimeric structure of bicyclo[1.1.0]butyl lithium ligated with TMEDA. Carbon=grey; nitrogen=blue; lithium=pink. Hydrogen atoms omitted for clarity.

**Figure 4:**
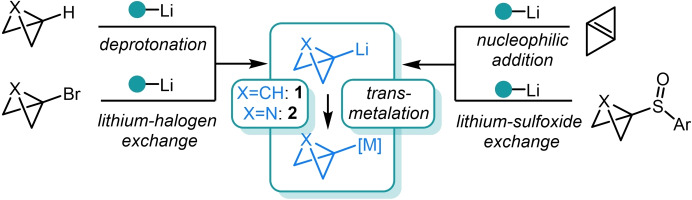
Synthetic methods to access (aza)bicyclo[1.1.0]butyl lithium intermediates **1** and **2**.

Interestingly, it was demonstrated by Muller and Pritchard that the amount of s‐character in carbon‐atomic orbitals involved in a C−H bond could be semi‐empirically determined from the observed ^13^C−^1^H NMR coupling constant (Figure 2).^[47]^ Again, the experimental value for this coupling constant (202 Hz) was found to lie in between that of ethylene (157 Hz) and ethyne (248 Hz).[^1^, ^47^] It is these anion stabilizing effects that allow the straightforward synthesis of metalated (aza)bicyclo[1.1.0]butanes, which can be most readily accessed through the deprotonation of the unusually acidic bridgehead C−H bond.[^48^, ^49^] This provides the opportunity to generate bridgehead functionalized bicyclic species without the need to install a synthetic handle in the starting materials. Substitution at this position is often targeted as the electronics of the bridgehead substituents can have a drastic effect on the reactivity of the central bond, for example electron withdrawing groups are routinely included to promote the addition of nucleophiles[^19^, ^20^, ^21^] and nucleophilic radicals.[^24^, ^25^, ^26^] Typically, strong organolithium bases (^n^BuLi/^s^BuLi) are employed in conjugation with chelating ligands such as tetramethylethylenediamine (TMEDA) to facilitate deprotonation.[^50^, ^51^] In addition to enhancing the basicity of the organolithium reagent by disfavoring lithium aggregates, this diamine ligand may also stabilize the resulting metalated intermediate. In 1973, Stucky and coworkers reported the first crystal structure of bicyclo[1.1.0]butyl lithium, which was shown to adopt a dimeric structure in the solid state in which each lithium atom is equally bonded to two bridgehead carbon atoms (Figure 3).^[52]^ It was later shown that this dimeric arrangement of the organolithium also predominates in solution. This was demonstrated by Seebach and coworkers through the analysis of the ^13^C−^6^Li NMR splitting pattern at low temperatures.^[53]^

(Aza)Bicyclo[1.1.0]butyl lithium species **1** and **2** (Figure 4) lie at the heart of the field of metalated bicycles due to their relative ease of formation and their ability to undergo facile transmetalation with a broad range of elements such as Mg, Cu, Sn, Zn, Ni, Fe and Au (see below). As well as deprotonation, lithium‐halogen exchange of the corresponding alkyl halide species has been demonstrated to be highly effective in accessing these valuable intermediates.^[40]^ Finally, nucleophilic addition to internal π‐bonds^[54]^ and lithium‐sulfoxide exchange[^51^, ^55^] have also been employed to a lesser extent. Although easily handled and crystalline solids, a notable drawback to using aryl sulfoxide precursors is the rapid equilibrium that ensues upon exposure to organolithium species which promotes the formation of the thermodynamically favored sp^2^ anion. This restricts its use to reactions that allow in situ lithiation conditions to trap out the desired organometallic reagent with the targeted electrophile before the detrimental effects of this equilibrium are observed.[^51^, ^55^, ^56^]

## 2 Rearrangements of Metalated Bicyclo[1.1.0]butanes

**Scheme 1:**
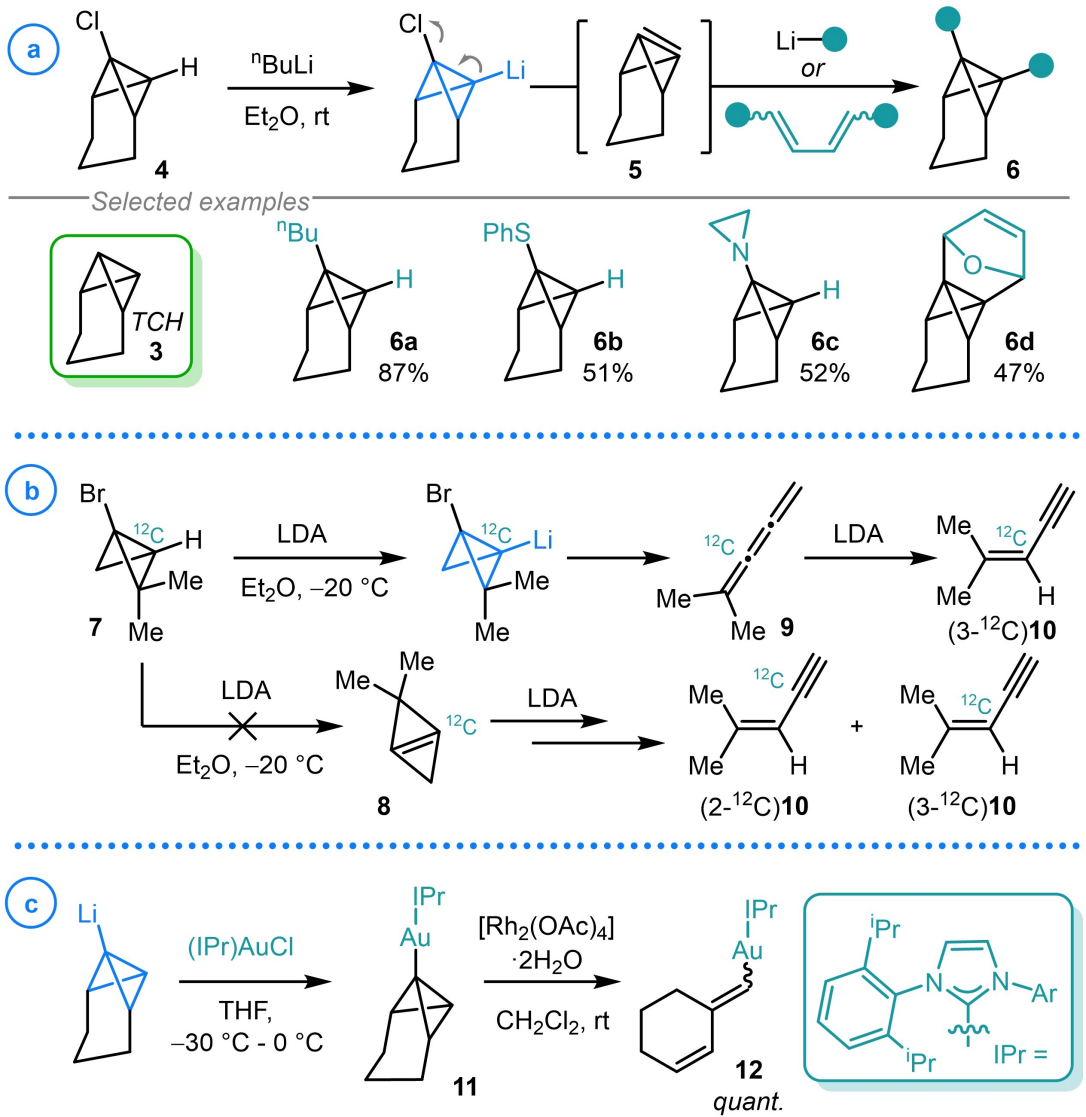
a) Szeimies’ synthesis and reactivity bicyclo[1.1.0]but‐1(3)‐ene fragment **5**. b) Isotopic labelling study of the rearrangement of lithiated 1‐bromobicyclo[1.1.0]butane **7**. c) Hashmi's synthesis and rearrangement of TCH‐gold(I) complex **11**.

The tricyclo[4.1.0.0^2,7^]heptane (TCH, **3**) scaffold has historically been employed as a readily prepared and easily handled analogue of bicyclo[1.1.0]butane, and assumed to be a valid reactivity model as the flexibility of the trimethylene bridge should not introduce significant strain into the system.^[57]^ In 1977 Szeimies and coworkers reported the intermediacy of the anti‐Bredt bicyclo[1.1.0]but‐1(3)‐ene fragment (**5**) via the β‐elimination of 1‐chloro‐TCH (**4**) upon deprotonation with ^n^BuLi (Scheme 1a).^[54]^ Intermediate **5** was demonstrated to react with a variety of different nucleophiles such as organolithiums (**6 a**),^[54]^ lithium thiolates (**6 b**),^[58]^ lithium amides (**6 c**)^[59]^ and 1,3‐dienes in [4+2] cycloaddition reactions (**6 d**).^[60]^ This provided rapid access to a series of unique scaffolds bearing the bicyclo[1.1.0]butane fragment by harnessing a species with even greater ring strain than that displayed by the products. However, in a similar study, the corresponding 1‐bromobicyclo[1.1.0]butane (**7**) was not observed to form the expected bicyclo[1.1.0]but‐1(3)‐ene intermediate (**8**) and instead spontaneously rearranged to 1,2,3 triene **9**, which underwent further base‐induced isomerization to ene‐yne **10** (Scheme 1b).^[61]^ β‐Elimination product **8** was ruled out using an isotopic labelling study which demonstrated that a single labelled product (3‐^12^C)**10** was obtained when 1‐C^12^ enriched **7** was employed. This clearly showed that symmetrical alkene **8** was not an intermediate on this pathway or, assuming no contribution from a kinetic isotope effect, a 1 : 1 mixture of (2‐^12^C)**10** and (3‐^12^C)**10** would be observed.

In 2018, Hashmi and coworkers reported the preparation and subsequent isomerization of the first example of a TCH‐gold(I) complex (**11**, Scheme 1c).^[62]^ By again exploiting the enhanced acidity of the bridgehead methine of TCH (**3**), deprotonation using ^n^BuLi could be followed by transmetalation with (IPr)AuCl to access the corresponding NHC bound TCH‐gold(I) species (**11**). Under a variety of different conditions, **11** was observed to undergo isomerization to the corresponding cyclohex‐2‐en‐1‐ylidenemethyl gold(I) complex (**12**). This was most effectively achieved with 5 mol % [Rh_2_(OAc)_4_]⋅2H_2_O which gave quantitative formation of **12** as a 1 : 1 ratio of E : Z isomers. Unfortunately, attempts to further derivatize this gold complex by hydrogenation or oxidation were unsuccessful and so the synthetic utility of this highly strained organogold complex is significantly limited.

## 3 Isotopic Labelling of Metalated Bicyclo[1.1.0]butanes

**Scheme 2:**
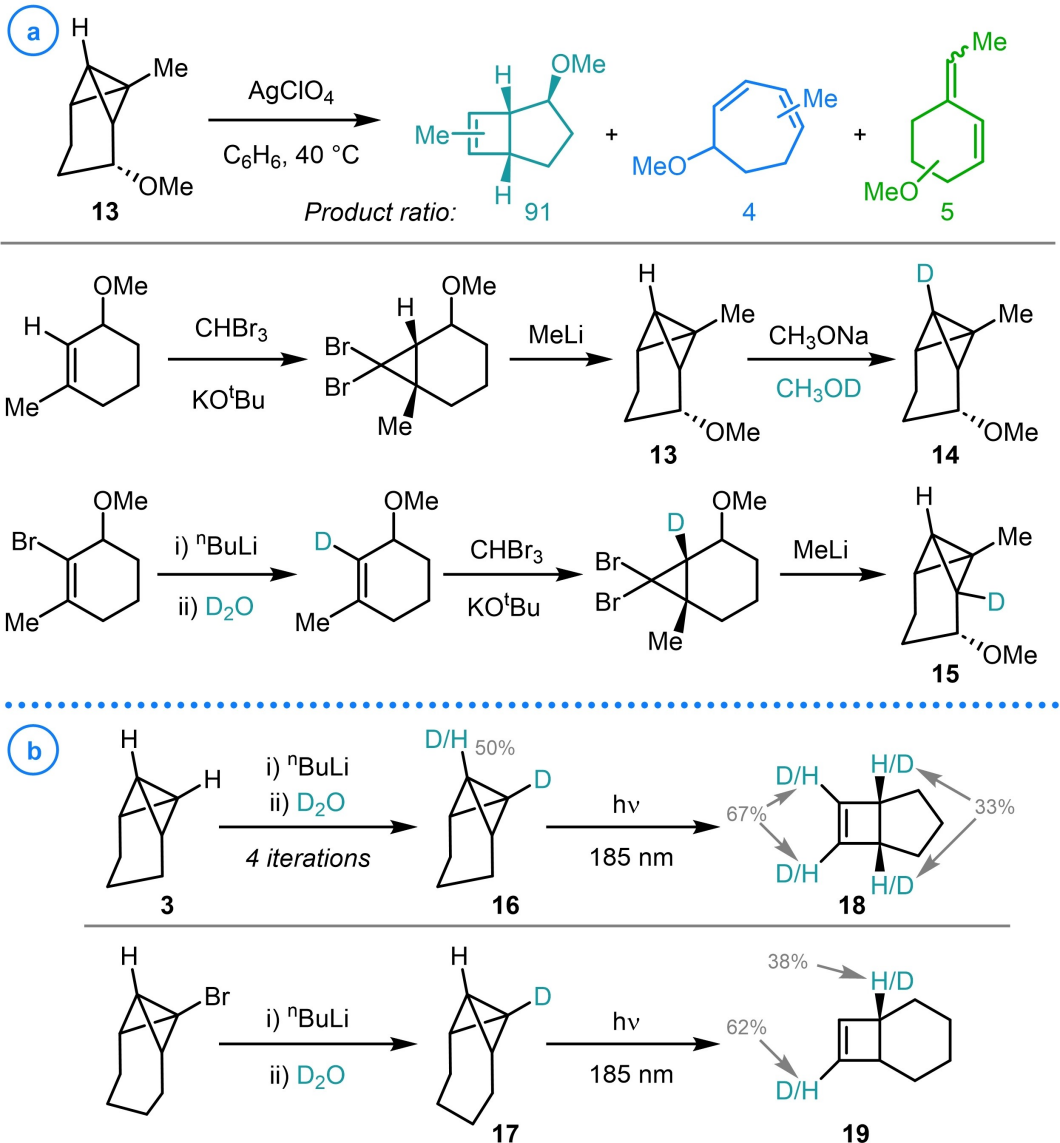
a) Paquette's study of the Ag(I)‐catalyzed rearrangement of TCH‐based structure **13**. b) Adam's isotopic labeling approach to probing the relationship between bicyclo[1.1.0]butane inter ring angle and the mechanism of light‐induced rearrangement. Degree of deuteration shown as a percentage of available deuterium.

The ability to access isotopically labelled adducts of bicyclobutane‐containing species has been vital in gaining insight into their structure, bonding and the reaction mechanisms of strain‐promoted transformations. In this regard, the deuteration of carbon–lithium bonds represents the most efficient and facile way to access isotope analogues at a late‐stage and using inexpensive sources of deuterium (namely D_2_O and MeOD). An example of this was exemplified by Paquette and coworkers in their study of the Ag(I)‐catalyzed rearrangement of TCH‐based structure **13** (Scheme 2a).^[63]^ In an effort to understand the product distribution and elucidate the mechanism of this isomerization process, a kinetic isotope effect study was undertaken using C7 and C2 deuterium labelled starting materials (**14** and **15**). Due to the acidity of the bridgehead position, deuteration of the C7 methine could be readily achieved through the subjection of the standard substrate to MeONa in MeOD. Conversely, C2 labelled substrate **15** could only be accessed after a revision of the synthetic route which necessitated the laborious installation of a bromide handle at an early stage in the synthesis.^[64]^

Similar deuterated bridgehead starting materials were also accessed by Adam and coworkers, who employed deprotonation and lithium‐halogen exchange conditions to access deuterated bicyclo[1.1.0]butane‐containing structures **16** and **17** respectively (Scheme 2b).^[65]^ These strained species were then used to probe the light‐induced rearrangements of tethered bicyclo[1.1.0]butanes to fused cyclobutenes (**18** and **19**), with the site and relative degree of deuteration indicating that various competing mechanisms were occurring at different rates for the two substrates. This data was instrumental in the establishment of the relationship between the bicyclo[1.1.0]butane inter ring angle and the mechanism of light‐induced rearrangement by employing substrates of differing tether length.

**Scheme 3:**
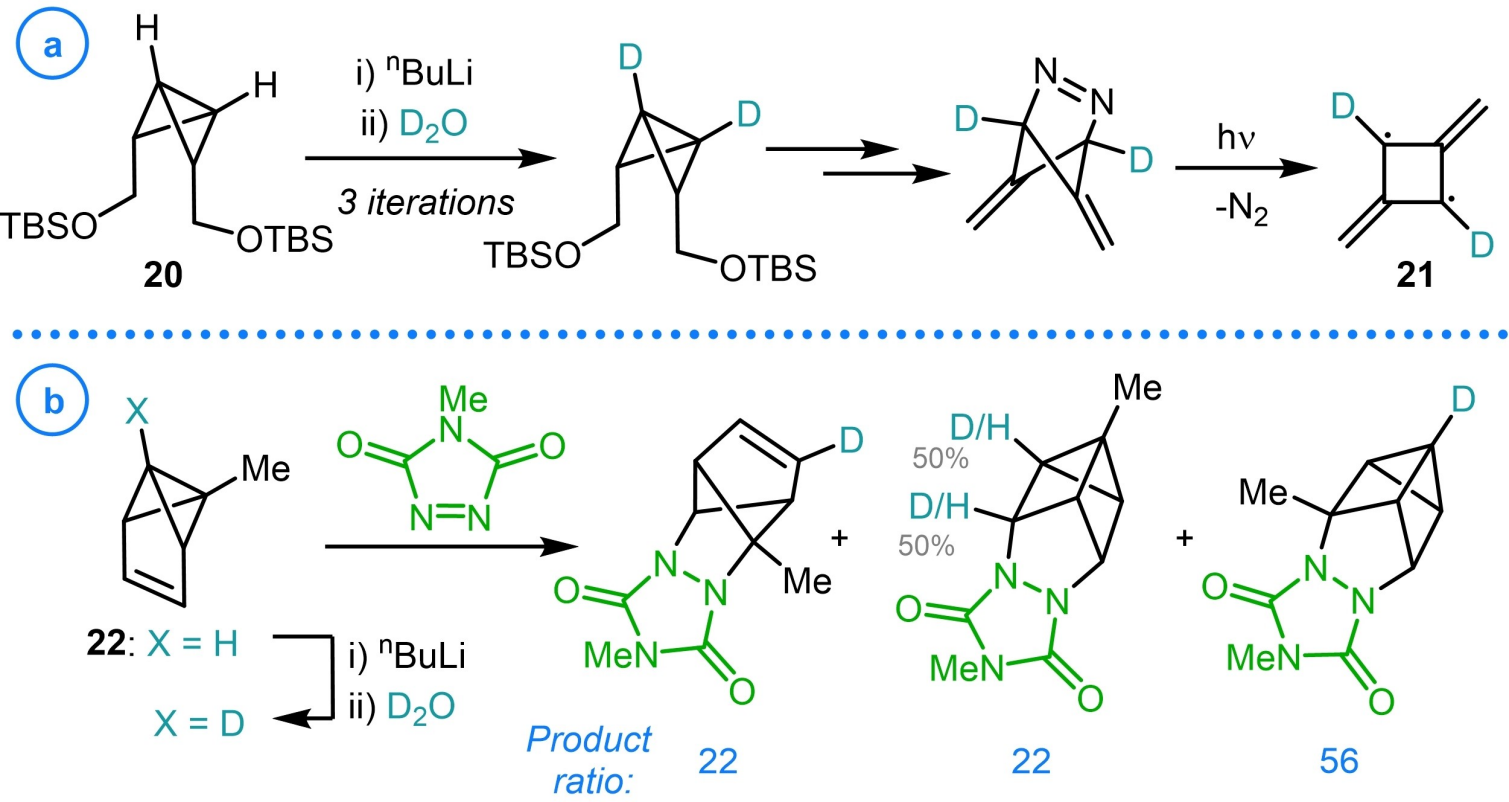
a) Snyder and Doughherty's synthesis of isotopically labelled biradical **21**. b) Christl's study of benzvalene cycloadditions reactions. Degree of deuteration shown as a percentage of available deuterium.

Snyder and Doughherty used a related strategy in their spectroscopic study of the non‐Kekulé isomer of benzene, 2,4‐dimethylene‐1,3‐cyclobutanediyl (Scheme 3a, **21**).^[66]^ Bicyclo[1.1.0]butane‐containing **20**, a precursor to the targeted biradical, was readily deuterated at both bridgehead positions providing access to an isotopically labelled analogue, which was instrumental for the characterization of **21** using EPR spectroscopy.^[67]^ In a study into the scope of the reactivity of benzvalene (**22**), Christl and coworkers also relied on the predictable deprotonation of these strained species to install a bridgehead deuterium label, whose presence in the resulting cycloaddition products gave insights into the complex mechanisms of the rearrangement process under investigation (Scheme 3b).[^68^, ^69^]

**Scheme 4:**
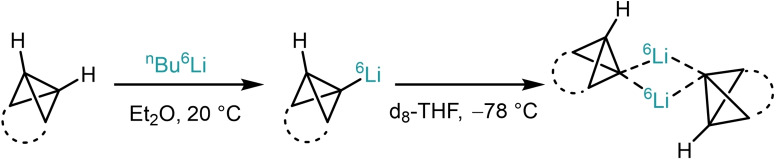
a) Szeimies’ and Seebach's synthesis of ^6^Li labelled bicyclo[1.1.0]butyl lithium.

An alternative strategy used to access isotopically labelled bicyclo[1.1.0]butane compounds is to generate the corresponding organolithium species using the less abundant lithium isotope, ^6^Li. This has been successfully performed by the groups of Szeimies and Seebach through the deprotonation of several bicyclobutanes with ^n^BuLi solution enriched in the light lithium isotope (Scheme 4).[^53^, ^70^] In both the aforementioned studies, these labelled organometallics were used to probe the extent of the molecule's aggregation in solution for mono‐ and dilithiated bicyclo[1.1.0]butanes using NMR spectroscopy. This ability to rapidly access isotopically labelled strained fragments in multiple ways has contributed significantly to the study of the unique properties of bicyclo[1.1.0]butane‐containing molecules.

## 4 Carbon‐Heteroatom Bond Formation

**Scheme 5:**
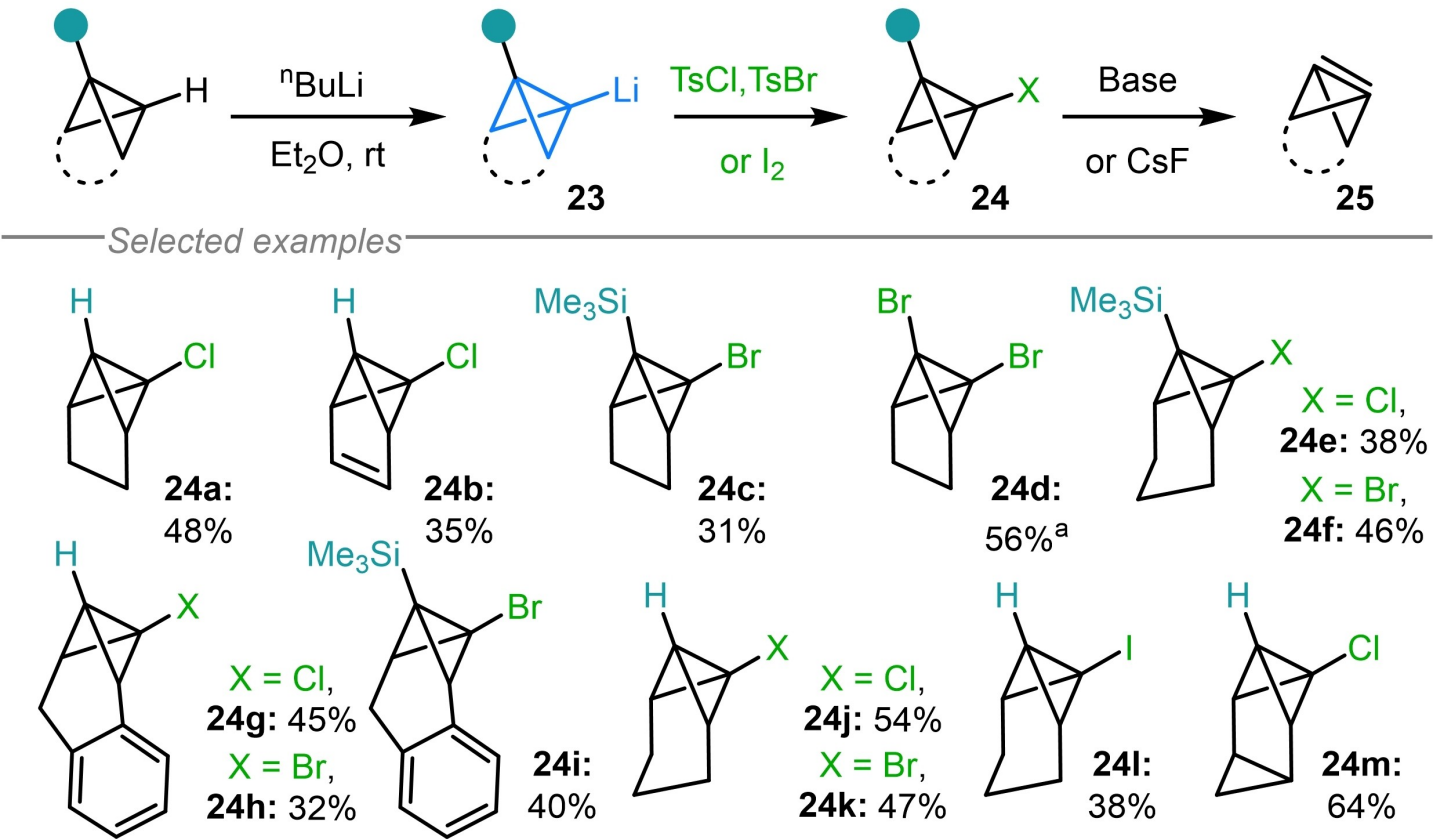
Szeimies’ synthesis of bicyclo[1.1.0]butyl halides **24**. ^a^ From doubly lithiated bicyclo[1.1.0]butane.

The potential use of bicyclo[1.1.0]butyl metal species as linchpin reagents is evidenced by their ability to forge carbon‐heteroatom bonds to install versatile functional handles on highly strained scaffolds. In their studies on the synthesis of the highly reactive bicyclo[1.1.0]but‐1(3)‐ene fragment (**25**), Szeimies and coworkers demonstrated, for the first time, the use of organolithium **23** as an intermediate in the synthesis of chloro‐, bromo‐ and iodo‐substituted bicyclo[1.1.0]butanes (**24**, Scheme 5).[^60^, ^71^, ^72^, ^73^, ^74^, ^75^, ^76^] This was achieved in moderate to low yields using TsCl, TsBr and iodine electrophiles respectively. The resulting bicyclo[1.1.0]butyl halides **24 a**–**24 m** were shown to eliminate upon exposure to base (or CsF in the case of silylated bicyclo[1.1.0]butanes) to access the targeted strained alkenes (**25**).^[77]^

**Scheme 6:**
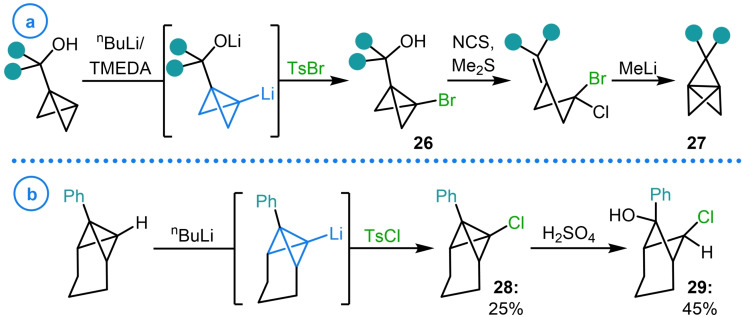
a) Szeimies’ bromination procedure in the synthesis of [1.1.1]propellane derivatives. b) Christl's synthesis of chloro‐TCH **28**.

In an unrelated study, Szeimies and coworkers employed the aforementioned bromination procedure to access halogenated bicyclo[1.1.0]butanes **26** that were used in the synthesis of [1.1.1]propellane derivatives **27** via a Corey–Kim‐type oxidation followed by an intramolecular [2+1] cycloaddition reaction (Scheme 6a).[^78^, ^79^] The same chlorination procedure was also exploited by Christl and coworkers during their study on the ring‐opening mechanism of bicyclo[1.1.0]butane‐containing molecules with electrophiles (Scheme 6b).^[80]^ Here, the incorporation of a chlorine atom in **28** was required to access cyclobutane **29**, whose spectroscopic data helped to verify the stereochemistry of other ring opened products under investigation. Although typically used to provide a synthetic handle upon ring‐opening, the installation of halogen atoms on strained scaffolds can also be used to enable site selective access to metalated bicyclo[1.1.0]butanes via lithium‐halogen exchange or magnesium insertion.^[74]^

**Scheme 7:**
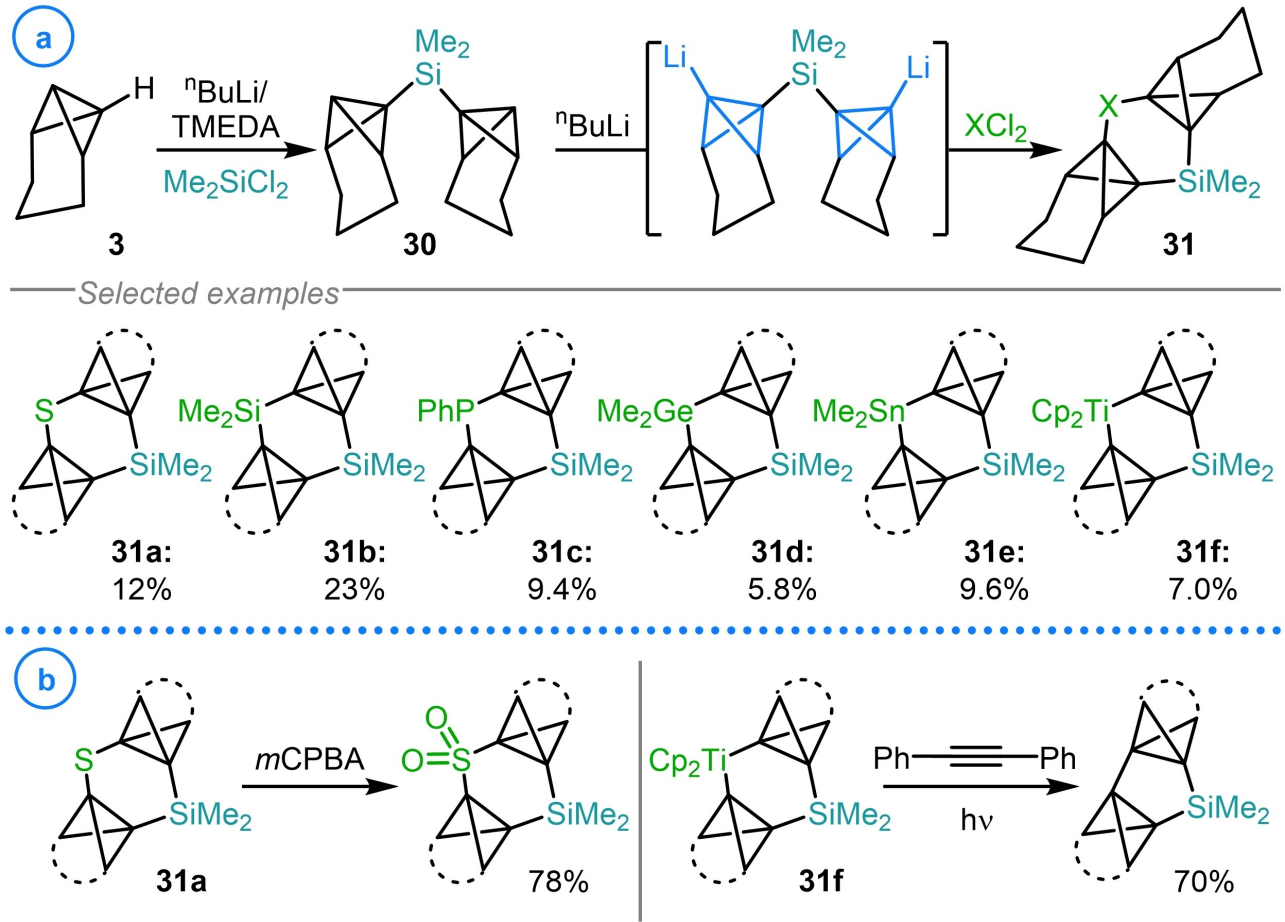
a) Szeimies’ carbon‐heteroatom bond formation using ‘pincer’ complex **30**. b) Examples of product derivatization.

The most common heteroatom‐based electrophiles that have been used as reaction partners with metalated bicyclo[1.1.0]butanes are silyl chlorides, exemplified by the work of Ando[^81^, ^82^] and Rücker.[^83^, ^84^] Although this type of reactivity has been demonstrated on numerous systems, the relative inertness of the resulting C−Si bond has limited the synthetic potential of such species. However, this reactivity was uniquely harnessed by Szeimies and coworkers in 1986 in an attempt to explore the scope of heteroatom‐based electrophiles that can react with metalated bicyclo[1.1.0]butanes (Scheme 7a).^[50]^ This was facilitated by ‘pincer’ complex **30**, synthesized via the reaction of lithiated TCH (**3**) and dimethyldichlorosilane.^[85]^ Compound **30** was then doubly deprotonated with ^n^BuLi/TMEDA before intramolecular cyclization was induced with a variety of dichloride electrophiles. In this regard, C−S, C−Si, C−P, C−Ge, C−Sn and C−Ti bonds were all generated in the formation of the corresponding [4.1.1]propellanes (**31 a**–**31 f**).^[50]^ However, the yields of this ring‐closure procedure were disappointingly low. According to the authors, this was a consequence of the lack of reaction dilution which resulted in the considerable production of polymeric material, significantly limiting reaction yield and impeding the isolation of the products. Despite this limitation, the synthetic utility of the products was briefly explored with sulfide **31 a** being oxidized to the corresponding sulfone with hydrogen peroxide and organotitanium **31 f** undergoing photochemical extrusion of titanocene, in the presence of diphenylacetylene, to deliver the desired ring contracted [3.1.1]propellane product in 70 % yield (Scheme 7b).

**Scheme 8:**
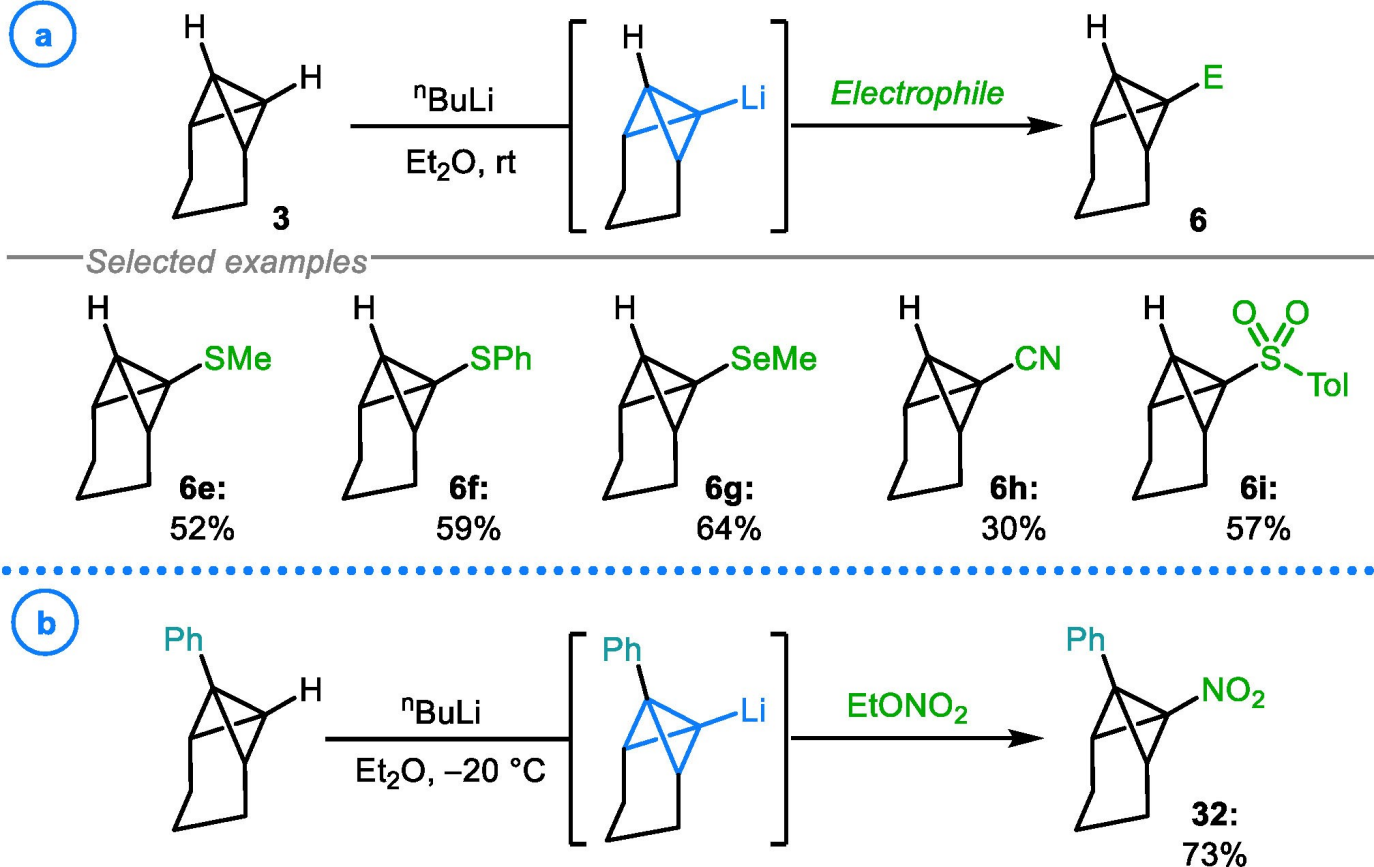
a) Szeimies’ scope of carbon‐heteroatom bond formation. b) Vasin's construction of C−N bonds on a TCH scaffold.

In one of the earliest studies into the reactions of lithiated TCH with various electrophiles, Szeimies and coworkers demonstrated that as well as halogenation, sulfuration (**6 e**–**6 f**) and selenation (**6 g**) could be achieved by employing the corresponding disulfides and diselenides (Scheme 8a).^[76]^ As well as this, bicyclo[1.1.0]butanyl cyanide **6 h** and sulfone **6 i** could be accessed using phenyl cyanate and 4‐methylbenzenesulfonyl fluoride, a strategy later utilized by Vasin and coworkers.^[86]^ Further contributions to the scope of heteroatom‐based electrophiles that react with metalated bicyclo[1.1.0]butanes were made by Vasin and coworkers, who successfully showed that C−N bonds could be forged through the use of ethyl nitrate, providing access to desired nitro compound **32** in 73 % yield (Scheme 8b).^[87]^

The formation of carbon‐boron bonds has also stimulated considerable interest for the ability of the resulting compounds to undergo a range of strain‐promoted transformations and is discussed separately in Section 5.4. Although several classes of heteroatom‐based electrophiles have been explored in the context of reactions with bridgehead metalated tricyclo[4.1.0.0^2,7^]heptanes, there is still a lack of general methods for such transformations. The generation of C−O and C−F bonds by this methodology is still unreported, and strategies that do not require the use of bifunctional ‘pincer’ complexes to facilitate carbon‐heteroatom bond formation are few in number.

## 5 Carbon‐Carbon Bond Formation

Arguably the most valuable transformation that metalated (aza)bicyclo[1.1.0]butanes can undergo are carbon‐carbon bond forming reactions to generate quaternary carbon centers. The unusually tight bond angle of the bridgehead carbons of metalated bicyclo[1.1.0]butanes significantly reduces the steric constraints placed upon the molecule compared to acyclic tertiary organometallics. This allows such species to participate in range of different nucleophilic addition reactions to sp^2^ and sp^3^ carbon‐based electrophiles to access synthetically valuable intermediates that are largely inaccessible by other methods. In this section, the various electrophiles that have been employed in such strategies will be examined and are discussed within the context of the unique reactivity that the subsequent products possess.

### 5.1 Alkyl halides and epoxides

**Scheme 9:**
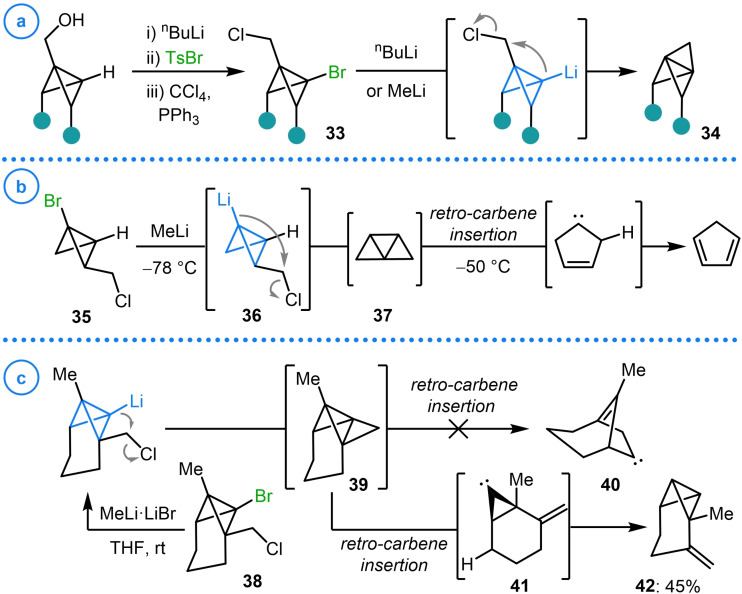
a) Szeimies, Kaszynski and Mazal's intramolecular cyclization strategy for [1.1.1]propellane synthesis. b) Wiberg's attempted synthesis of **37**. c) Observations made by Szeimies and coworkers during the synthesis of **39**.

The intramolecular S_N_2 displacement of an alkyl halide by metalated bicyclo[1.1.0]butanes has proved to be a reliable annulation method for the construction of scaffolds with an even greater degree of inherent ring strain than that displayed by bicyclo[1.1.0]butane and its analogues. An example of this reactivity, demonstrated by the groups of Szeimies,[^88^, ^89^] Kaszynski^[90]^ and Mazal,^[91]^ showed that the intramolecular substitution of primary alkyl chlorides (**33**), by employing organolithium reagents to facilitate lithium‐halogen exchange, could deliver [1.1.1]propellanes **34** (Scheme 9a).

The C2 substituted isomer of this cyclization precursor (**35**) was subjected to similar ring‐closure conditions by Wiberg and coworkers (Scheme 9b).^[92]^ Although the targeted tricyclo[2.1.0.0^1,3^]pentane structure (**37**) was not directly observed, the authors state that the formation of cyclopentadiene is evidence for the intermediacy of **37**, which decomposes above −50 °C via a retro‐carbene insertion and subsequent 1,2 C−H shift. In an effort to increase the lifetime of this highly strained species, Szeimies and coworkers performed the same intramolecular alkylation on 6‐methyltetracyclo[4.2.0^1,7^.0^5,7^]octane **38**.^[93]^ They hypothesized that retro‐carbene insertion would be energetically less favorable as it would require the formation of an anti‐Bredt olefin (**40**) and so should extend the lifetime of this [1.1.1]propellane isomer, allowing spectroscopic observation (Scheme 9c). However, the observed formation of **42** as the sole reaction product suggests an alternative retro‐carbene insertion pathway is possible to form intermediate **41** which can then undergo C−H insertion to form the exocyclic alkene‐containing bicyclo[1.1.0]butane **42**. It must be noted that in all the above cases, the bridgehead bromine atom is also installed from the corresponding metalated bicyclo[1.1.0]butane intermediate under the same conditions shown in Scheme 5.

**Scheme 10:**
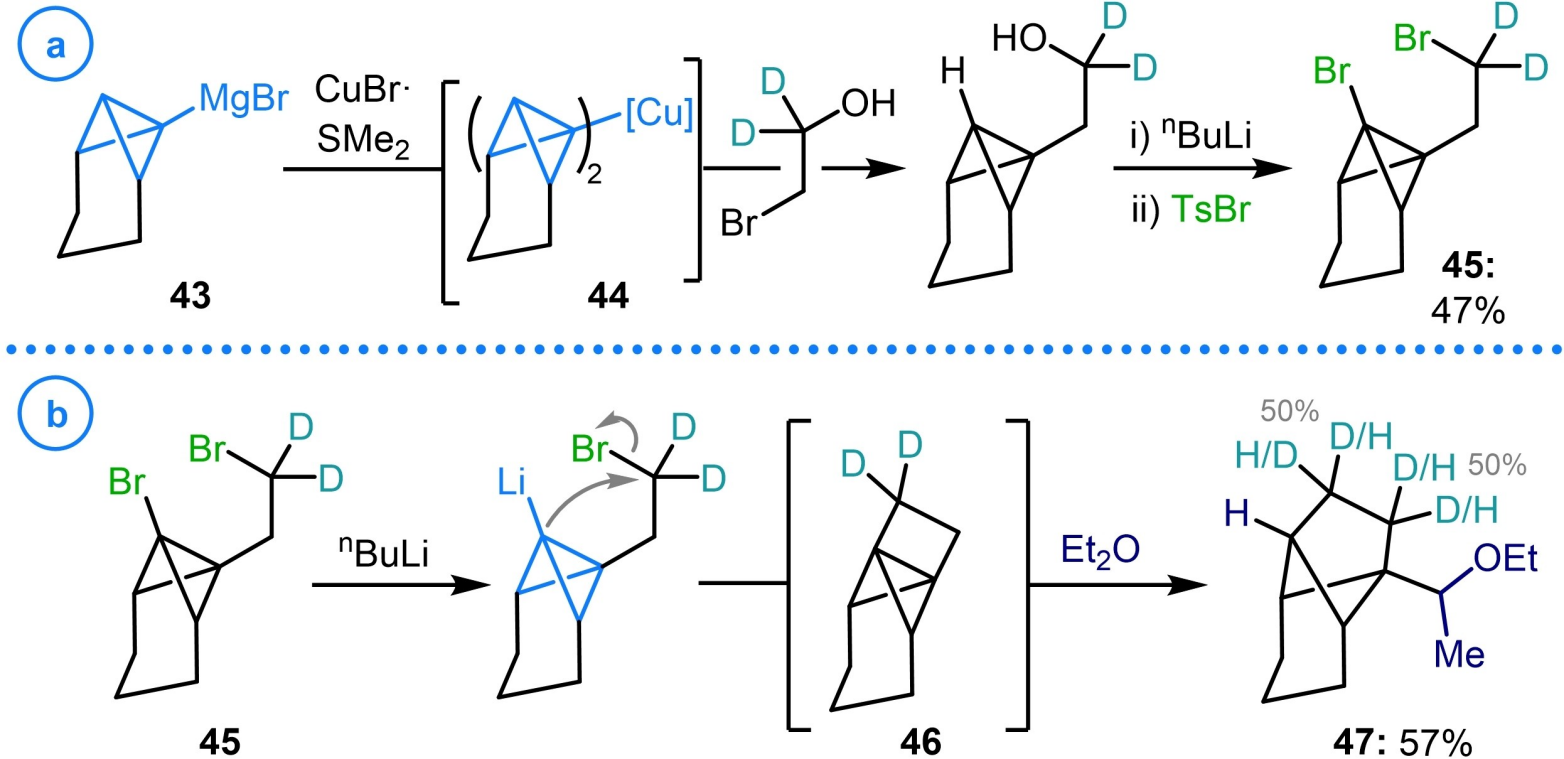
a) Szeimies’ organocuprate (**44**) addition to 2‐bromoethanol‐d_2_. b) Application of **45** to the synthesis of [2.1.1]propellane **46**.

When Szeimies extended this strategy to the synthesis of [2.1.1]propellanes (**46**), not only was intramolecular alkylation via the corresponding lithiated bicyclo[1.1.0]butane utilized to enact the ring‐closing step, but intermolecular alkylation was employed to access propellane precursor **45** (Scheme 10a).^[94]^ This was achieved via the generation of Gilman reagent **44** from 1‐tricyclo[4.1.0.0^2,7^]heptylmagnesium bromide (**43**) and catalytic CuBr⋅SMe_2_ before the addition of 2‐bromoethanol‐d_2_. The deuterium labelled bridge was used to provide evidence for the formation of [2.1.1]propellane **46**, as the target species was not observed upon ring‐closure (Scheme 10b). The even distribution of deuterium atoms in ether addition product **47**, the only discernable compound, provides evidence that this species arises from a (pseudo)symmetrical intermediate (presumably **46**) via a radical addition process.

**Scheme 11:**
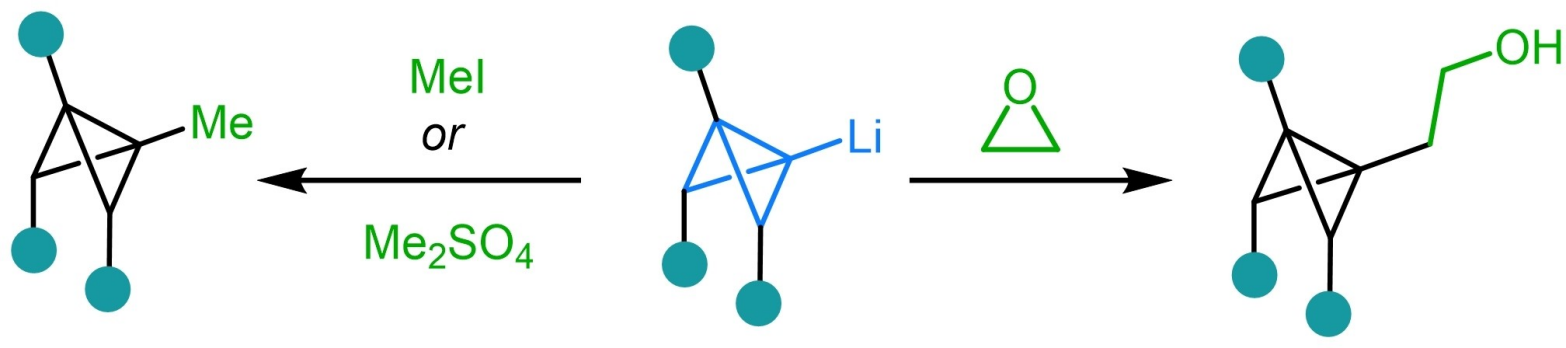
Reaction of lithiated bicyclo[1.1.0]butanes with methylating agents and epoxides.

Whilst, in the case above, transmetalation from the organomagnesium compound to the corresponding Gilman reagent was necessary to facilitate this nucleophilic displacement, it has been shown that alkylation can readily occur directly from lithiated bicyclo[1.1.0]butanes. Both methyl iodide^[93]^ and dimethyl sulfate^[95]^ have been employed for the methylation of substituted bicyclo[1.1.0]butanes, although this is typically used to protect a potentially reactive site (Scheme 11). On the other hand, the reaction of lithiated bicyclo[1.1.0]butanes with epoxides has been demonstrated to be an effective way to both alkylate the bridgehead position and to install a highly versatile alcohol handle that can be engaged in subsequent reactions.[^96^, ^97^]

**Scheme 12:**
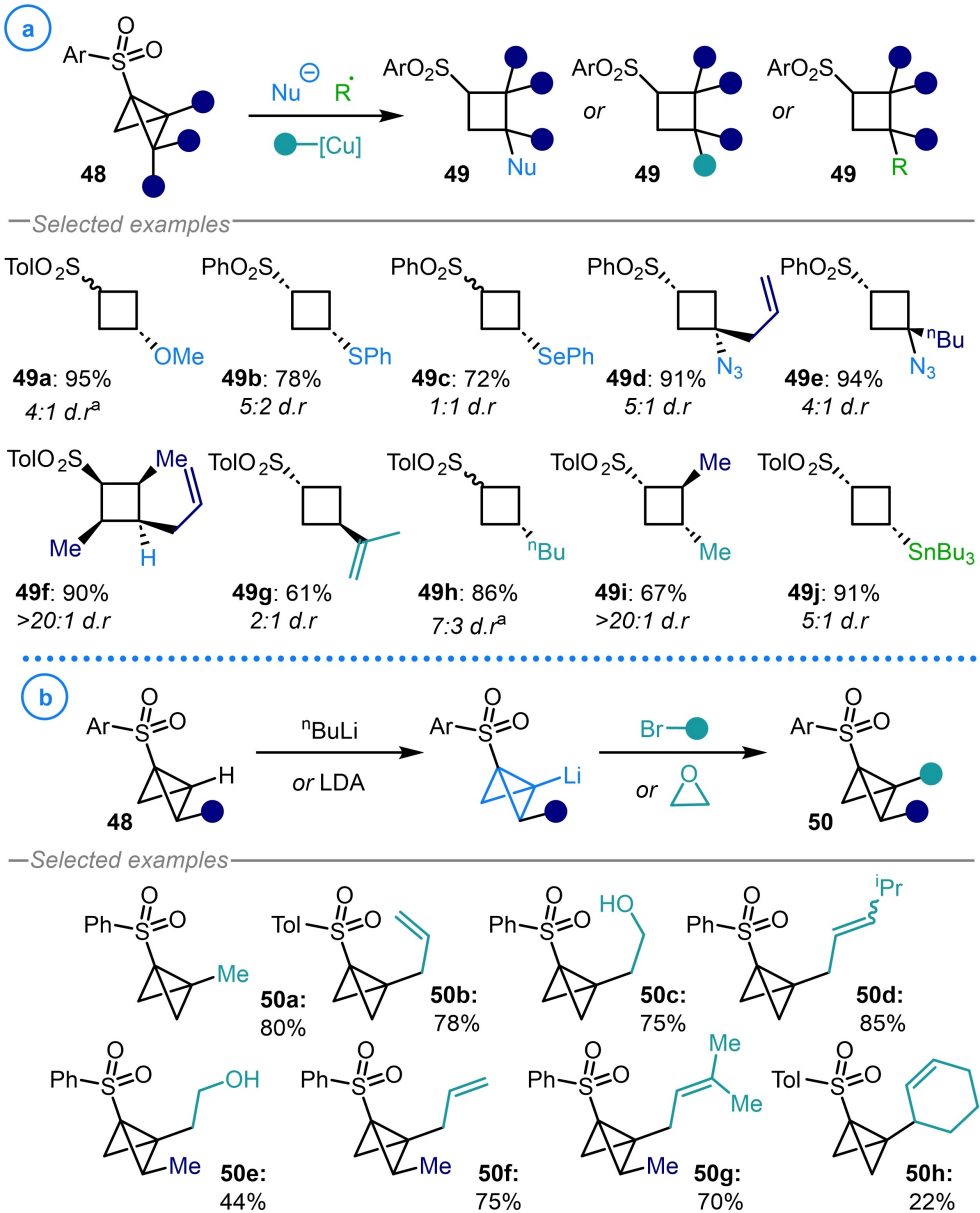
a) Gaoni's ring‐opening reactions of 1‐(arylsulfonyl) bicyclo[1.1.0]butanes (**48**). b) Umpolung reactivity facilitated by bridgehead deprotonation and subsequent alkylation. ^a^ Identity of major diastereomer unknown.

In an extensive study on the reactivity of 1‐(arylsulfonyl)bicyclo[1.1.0]butanes **48**, Gaoni and coworkers demonstrated that these electron deficient species display similar reactivity to unsaturated carbonyl compounds.^[98]^ In this regard, it was shown that **48** can act as a Michael/Giese acceptor, allowing the addition of heteroatom‐based nucleophiles (**49 a**–**49 e**), hydride reductants (**49 f**), organocopper reagents (**49 g**–**49 i**) and nucleophilic radicals (**49 j**) to the bridgehead carbon (Scheme 12a).^[99]^ However, it was also revealed that umpolung reactivity could be achieved through deprotonation of the bridgehead C−H bond with ^n^BuLi to provide a species with nucleophilic character at the C3 position (Scheme 12b).^[48]^ This intermediate was then alkylated with allylic and homo‐allylic alkyl bromides, as well as ethylene oxide, to access 3‐substituted 1‐(arylsulfonyl) bicyclo[1.1.0]butanes (**50 a**–**50 h**) which could participate in further ring‐opening reactions.

### 5.2 Aldehydes, ketones, and imines

**Scheme 13:**
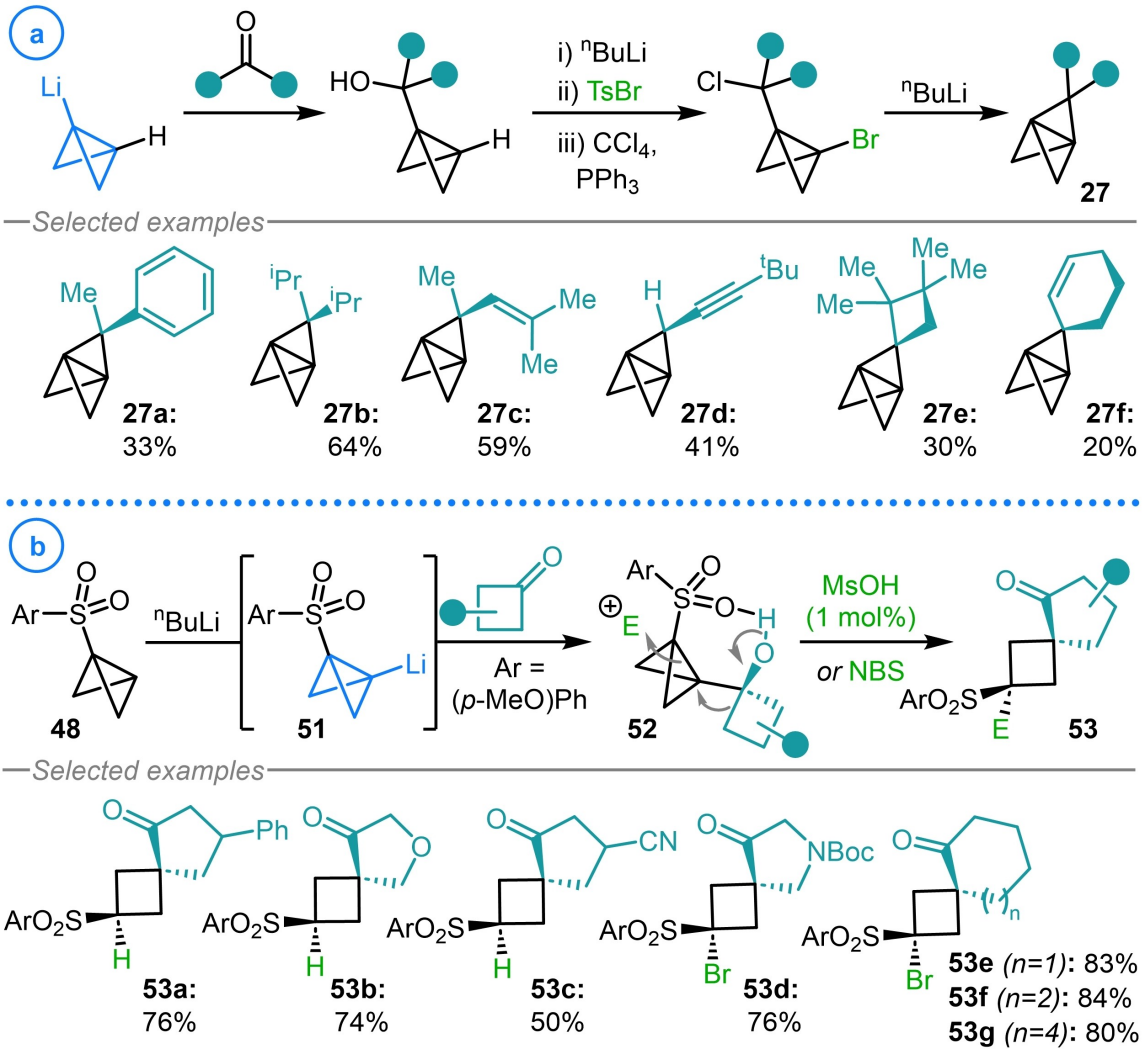
a) Szeimies’ aldehyde/ketone scope in the synthesis of [1.1.1]propellanes (**27**). b) Wipf's strain‐release semi‐pinacol rearrangement of bicyclo[1.1.0]butane carbinols (**52**).

The predictable reactivity of metalated (aza)bicyclo[1.1.0]butanes with carbonyl and imine electrophiles has become an invaluable tool for the installation of functional handles on strained scaffolds. In the syntheses of [1.1.1]propellanes (**27**) via intramolecular alkylation (see Section 5.1), the alkyl halide tethers are typically installed via the addition of lithiated bicyclo[1.1.0]butane species to aldehydes and ketones followed by an Appel reaction to install the chlorine atom (Scheme 13a).^[78]^ The late‐stage incorporation of aldehydes and ketones provides a desirable point of variation, which is readily exploited due to the commercial availability of these carbonyl compounds, to provide a unique method to access substituted [1.1.1]propellanes (**27 a**–**27 f**). In 1985, Gaoni and coworkers demonstrated that 1‐(arylsulfonyl)bicyclo[1.1.0]butanes **48**, once lithiated, readily undergoes 1,2‐nucleophilic addition reactions with a series of aldehydes and ketones.[^48^, ^99^] However, the synthetic potential of these bicyclo[1.1.0]butane carbinol products was not further explored. However, in recent years, considerable advances have been made in utilizing the products of these reactions for the synthesis of novel scaffolds. In 2021, Wipf and coworkers reported the addition of the same 1‐(arylsulfonyl) bicyclo[1.1.0]butane organolithium species (**51**) to cyclic ketones to generate tertiary alcohols **52** (Scheme 13b).^[100]^ These compounds, upon electrophilic activation of the bicyclic system with catalytic acid, were shown to undergo a highly diastereoselective strain‐promoted semi‐pinacol rearrangement to generate the corresponding spiro[3.4]octanes (**53 a**–**53 c**). The 1,2‐migration reaction could also be induced by *N*‐bromosuccinimide (NBS) to install a functional handle for further derivatization (**53 d**–**53 g**). These reactions highlight the potential of metalated bicyclo[1.1.0]butanes to act as carbenoids that react as nucleophiles in the first instance, whilst still retaining the electrophilic character of the bridgehead carbon for subsequent ring‐opening reactivity.

**Scheme 14:**
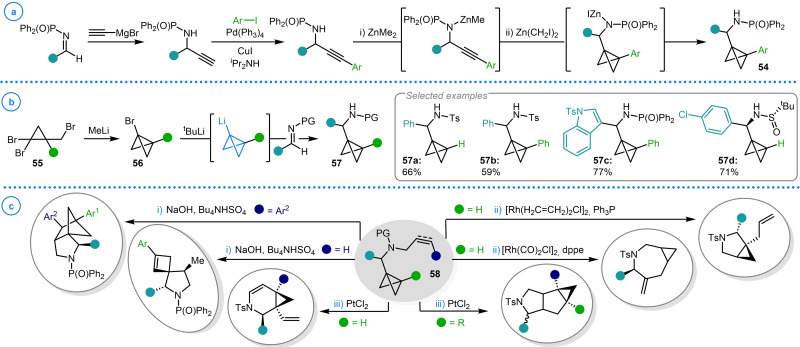
Wipf's study into the synthesis and reactivity bicyclo[1.1.0]butyl *N*‐allyl/propargylamines **58**. a) Simmons‐Smith cyclopropanation cascade reaction. b) Addition of bicyclo[1.1.0]butyl lithium to imines. c) Bicyclo[1.1.0]butyl *N*‐allyl/propargylamines in the divergent synthesis of cyclic scaffolds.

Another area within this field pioneered by the work of Wipf and coworkers is the addition of metalated bicyclo[1.1.0]butanes to imines. Although bicyclo[1.1.0]butyl amines (**54**) had been previously synthesized via Simmons–Smith cyclopropanation cascade reactions (Scheme 14a), a more general synthetic pathway was sought to broaden the scope of the potential products and improve the modularity of the procedure for greater substrate diversity.^[101]^ This was realized by utilizing dibromocyclopropylmethyl bromides (**55**) which, upon lithium‐halogen exchange with MeLi, gave access to bromo‐bicyclo[1.1.0]butanes **56** (Scheme 14b).^[102]^ This could then be reacted in situ with ^t^BuLi to enact another lithium‐halogen exchange event to deliver metalated bicyclo[1.1.0]butanes to which a variety of imine electrophiles could be added. Over a series of studies spanning a decade, a broad range of bicyclo[1.1.0]butyl alkylamines, including *N*‐tosyl, *N*‐diphenylphosphinoyl, and sulfinyl imine derivatives (**57 a**–**57 d**) were synthesized and subsequently allylated/propargylated.^[4c]^

The resulting bicyclo[1.1.0]butyl *N*‐allyl/propargylamines (**58**) were demonstrated to possess a broad reactivity profile, undergoing a variety of pericyclic and metal‐catalyzed reactions. These include intramolecular ene and [2+2] cycloaddition reactions using bicyclo[1.1.0]butyl *N*‐allyl amines (Scheme 14ci),[^102^, ^103^, ^104^] ligand controlled Rh(I)‐catalyzed cycloisomerizations to give cyclopropane‐fused pyrrolidines and azepines (Scheme 14cii),^[105]^ and divergent Pt(II)‐catalyzed rearrangements of bicyclo[1.1.0]butyl *N*‐propargyl amines (Scheme 14ciii).^[4c]^ Mechanistic discussions of the different reaction regimes that have been explored within the context of this study is beyond the scope of the present review and has been succinctly summarized in Ref. [4c].

**Scheme 15:**
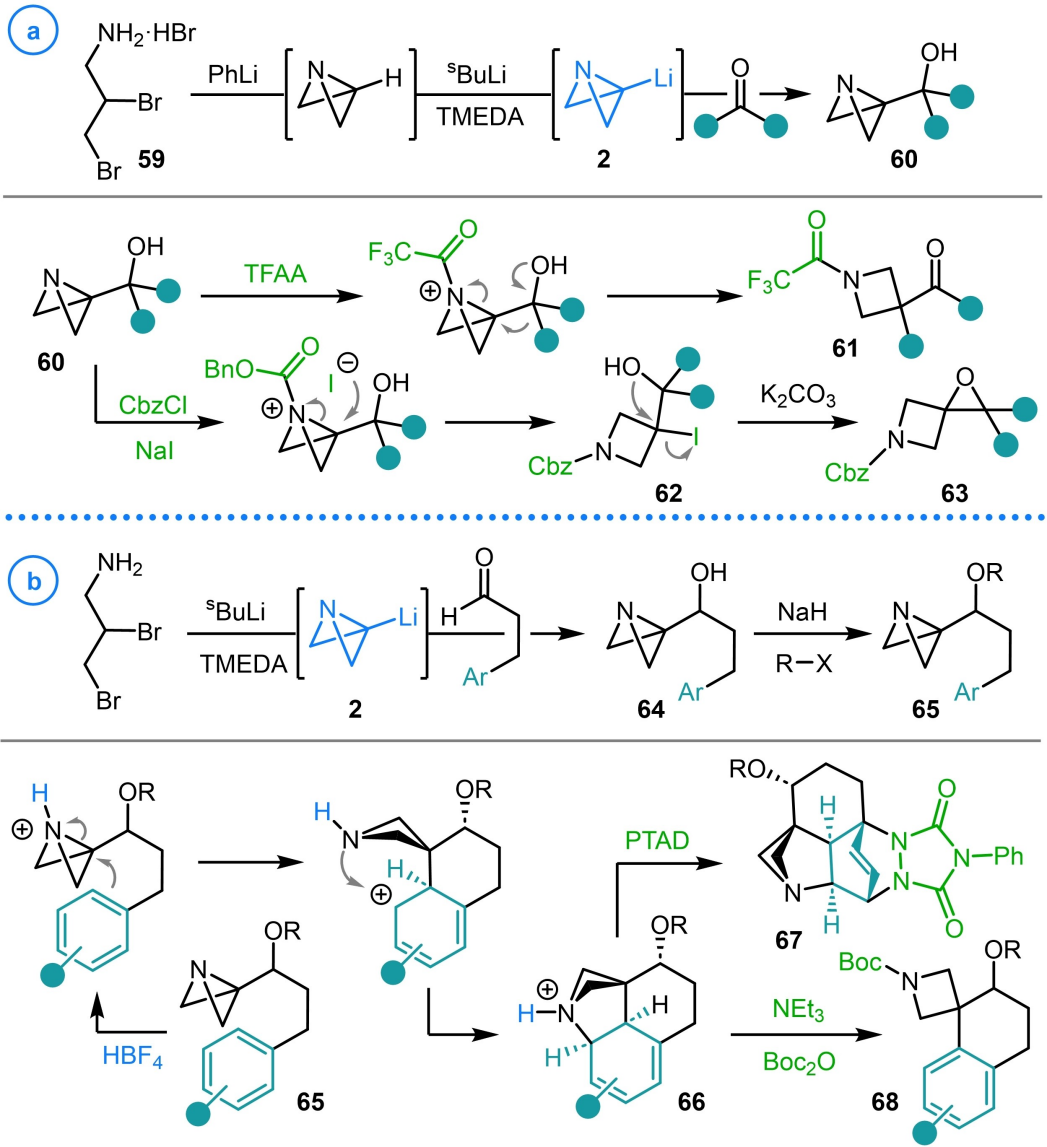
Addition of azabicyclo[1.1.0]butyl lithium to aldehydes and ketones. a) Aggarwal's strain‐release semi‐pinacol rearrangement of azabicyclo[1.1.0]butane carbinols. b) Divergent reactivity in Aggarwal's Friedel‐Crafts‐type intramolecular spirocyclization reaction.

In contrast to bicyclo[1.1.0]butanes, there is a dearth of studies that employ the metalated heterocyclic analogue azabicyclo[1.1.0]butane as a key intermediate. This is more a representation of the lack of attention that this fragment has received rather than a reflection of its reactivity profile which, when explored, has been demonstrated to react with a similarly broad range of electrophiles.^[33]^ An example of the unique reactivity that intermediates accessed from metalated azabicyclo[1.1.0]butanes can display was reported by Aggarwal and coworkers in 2021 (Scheme 15a).^[106]^ The intermediates in question, azabicyclo[1.1.0]butyl carbinols (**60**), could be rapidly synthesized in a single step via the addition of azabicyclo[1.1.0]butyl lithium (**2**) to a range of aldehydes and ketones. The rapid assembly of **2** was achieved from dibromoamine **59**, in which two consecutive nucleophilic displacement reactions assemble the aza‐bicycle, before a final deprotonation of the bridgehead C−H bond with ^s^BuLi/TMEDA delivers the carbenoid fragment. In a study performed concurrently with that reported by Wipf and coworkers (see Scheme 13),^[100]^ these azabicyclo[1.1.0]butyl carbinols (**60**) were demonstrated to, upon trifluoroacetylation of the tertiary amine, undergo a strain‐promoted semi‐pinacol rearrangement and deliver keto 1,3,3‐substituted azetidines (**61**).^[106]^ Alternatively, treatment of the same intermediates with benzyl chloroformate in the presence of NaI led to iodohydrin intermediates **62** which could generate spiroepoxy azetidines (**63**) in a base‐promoted ring‐closure reaction.

In a later study, Aggarwal and coworkers again harnessed the predictable reactivity between **2** and aldehydes to access aryl tethered azabicyclo[1.1.0]butanes **64** (Scheme 15b).^[107]^ In this case, the semi‐pinacol rearrangement of intermediate **64** represents an unproductive side reaction which necessitated the protection of the alcohol functionality via alkylation or silylation. The protected ABB‐carbinol products were also demonstrated to participate in divergent reactivity upon electrophilic activation of the aza‐bicycle. Protonation of **65** and subsequent strain‐release‐driven Friedel–Crafts‐type intramolecular spirocyclization, delivered a Wheland intermediate that could be trapped by the lone pair of the adjacent azetidine nitrogen, delivering diene‐containing azabicyclo[2.1.1]hexane intermediate **66**. This dearomatized species, formed exclusively as a single diastereomer, could be either rearomatized to azetidine spirotetralin **68** or stereoselectively trapped as Diels–Alder adduct **67** in a single step. Both these studies highlight how the carbenoid‐like reactivity of metalated azabicyclo[1.1.0]butanes can be used for the rapid assembly of functionalized azetidines.

**Scheme 16:**
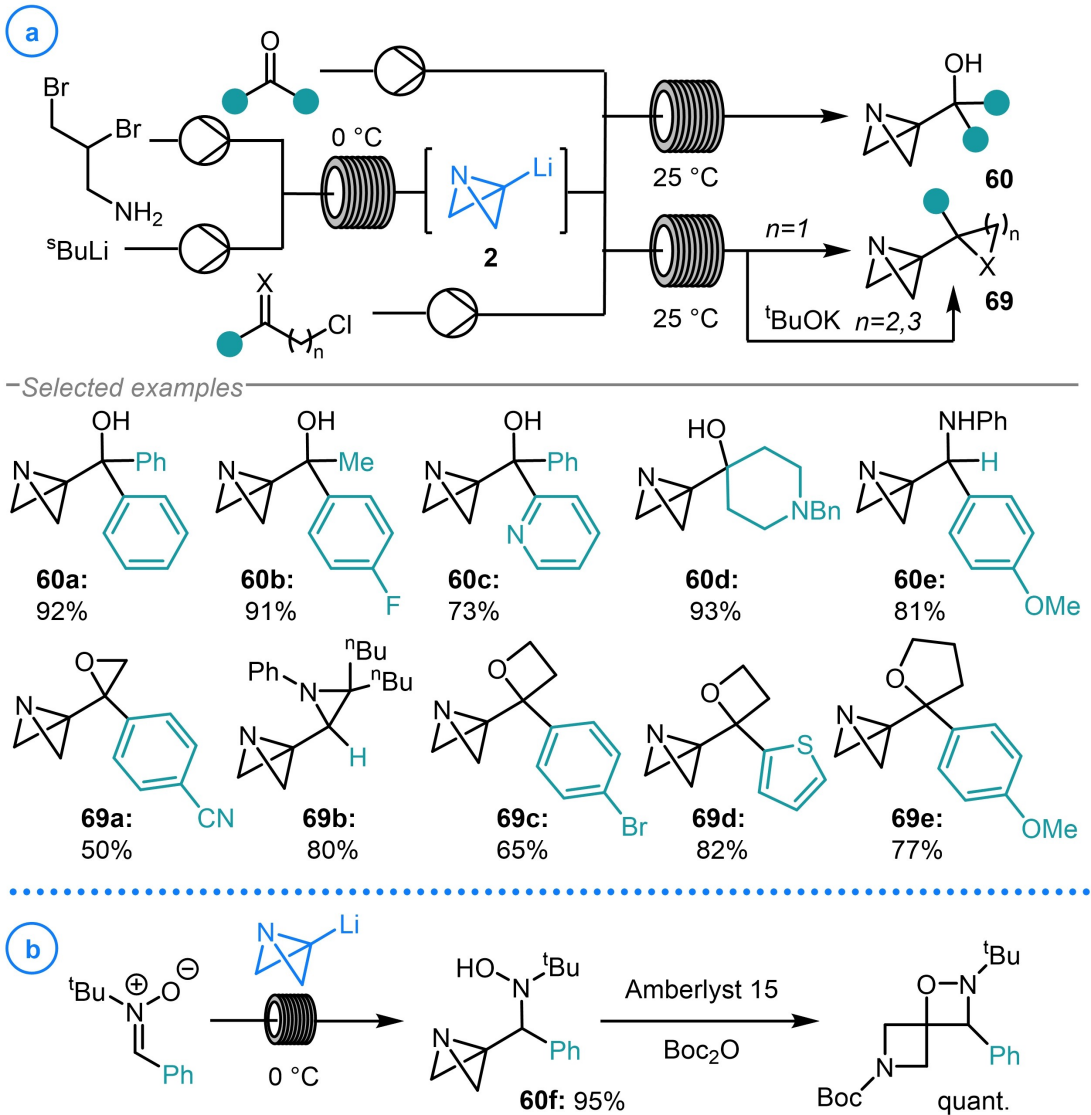
Luisi's generation of azabicyclo[1.1.0]butyl lithium (**2**) using flow chemistry and its subsequent reactivity with carbonyl‐based electrophiles.

In 2021, Luisi and coworkers reported the first continuous flow protocol for the generation of azabicyclo[1.1.0]butyl lithium **2**, representing a considerable advancement in the synthesis of bicyclo[1.1.0]butyl organometallics (Scheme 16a).^[108]^ The benefits of utilizing flow chemistry compared to the analogous batch process are numerous and include improved scalability, safer handling of pyrophoric materials and the ability to perform such a synthesis without the need for cryogenic temperatures. The assembly of **2** could also be telescoped with its subsequent reaction with a variety of electrophiles, including aldehydes, ketones and imines to provide access to broad range of strained compounds (**60 a**–**60 e**). Following on from this, a similar flow protocol was later used for the synthesis of azabicyclo[1.1.0]butanes bearing heterocycles (**69**).^[109]^ This was achieved through the continuous flow generation of azabicyclo[1.1.0]butyl lithium **2** which was then intercepted with α‐, β‐, and γ‐haloalkylketones/imines. The resulting intermediates can either spontaneously cyclize (n=1) or undergo base‐promoted cyclization from the corresponding chlorohydrin‐type intermediate in a subsequent step (n=2,3) to deliver products **69 a**–**69 e**. Additionally, the group demonstrated that azabicyclo[1.1.0]butyl lithium could be coupled with an imine N‐oxide to generate highly strained hydroxylamine adduct **60 f** (Scheme 16b). This species was subsequently subjected to acidic conditions to induce spirocyclization. Whilst currently representing the only example of such a protocol, this work is expected to inspire further research into the use of flow chemistry to modulate the synthesis and reactivity of metalated bicyclo[1.1.0]butanes.

**Scheme 17:**
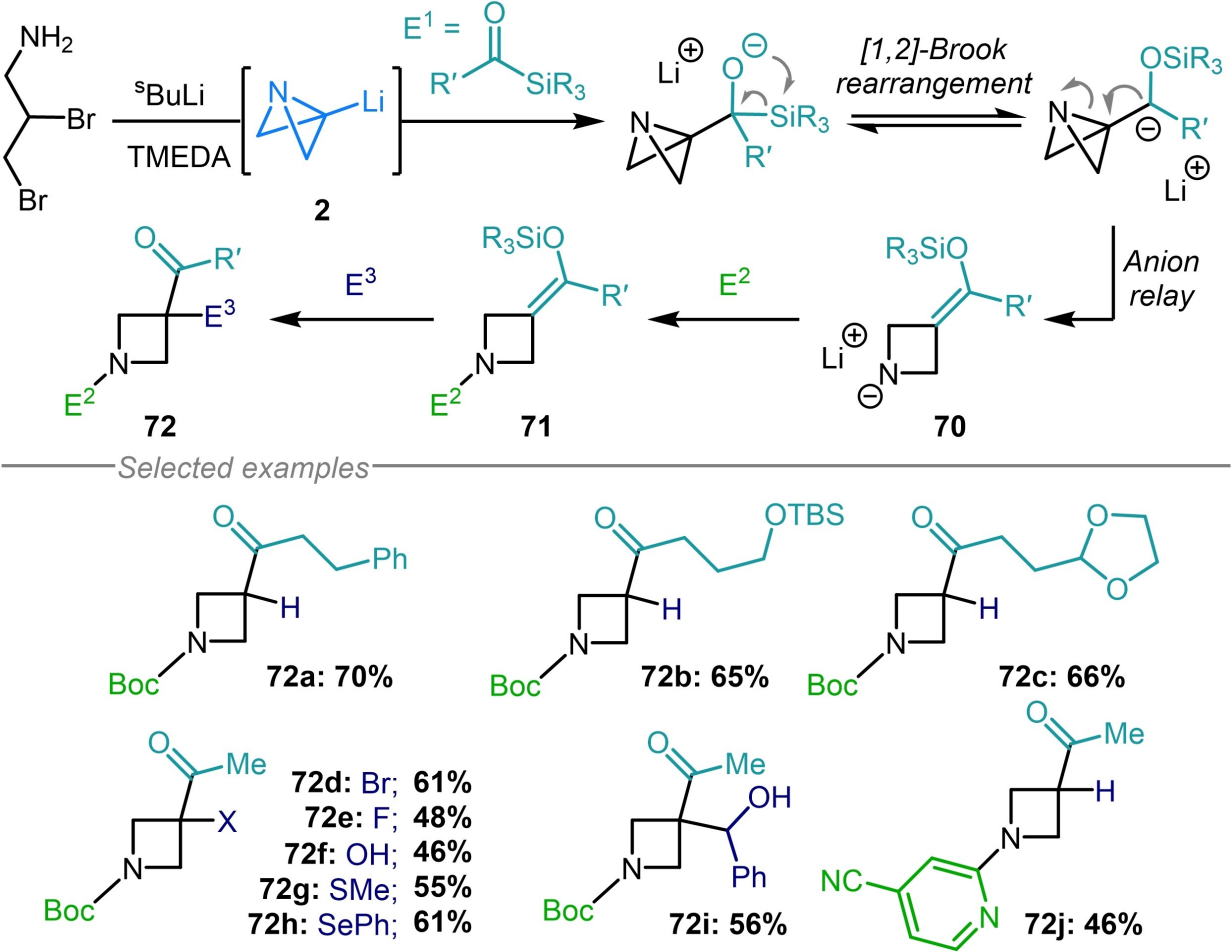
Aggarwal's four‐component [1,2]‐Brook rearrangement/strain‐release‐driven anion relay reaction.

Finally, Aggarwal and coworkers reported a multicomponent approach to azetidine synthesis which harnesses the inimitable reactivity of azabicyclo[1.1.0]butyl lithium (Scheme 17).^[110]^ This protocol involved the coupling of **2** with acyl silanes which, upon [1,2]‐Brook rearrangement and anion collapse to open the strained bicycle, delivered silyl enol ether **70**. This intermediate could then react with an electrophile at nitrogen and, after aqueous workup, with a further electrophile at the silyl enol ether fragment. The rapidity of this reaction was highlighted by the use of in situ infra‐red spectroscopy, which demonstrated that the entire synthesis of **71** could be achieved in just 45 minutes, with the synthesis of **2** being complete within the time taken for ^s^BuLi addition. Using this four‐component methodology, a variety of diverse 1,3,3 azetidines were assembled (**72 a**–**72 j**).

### 5.3 Esters, amides, chloroformates and carbon dioxide

**Scheme 18:**
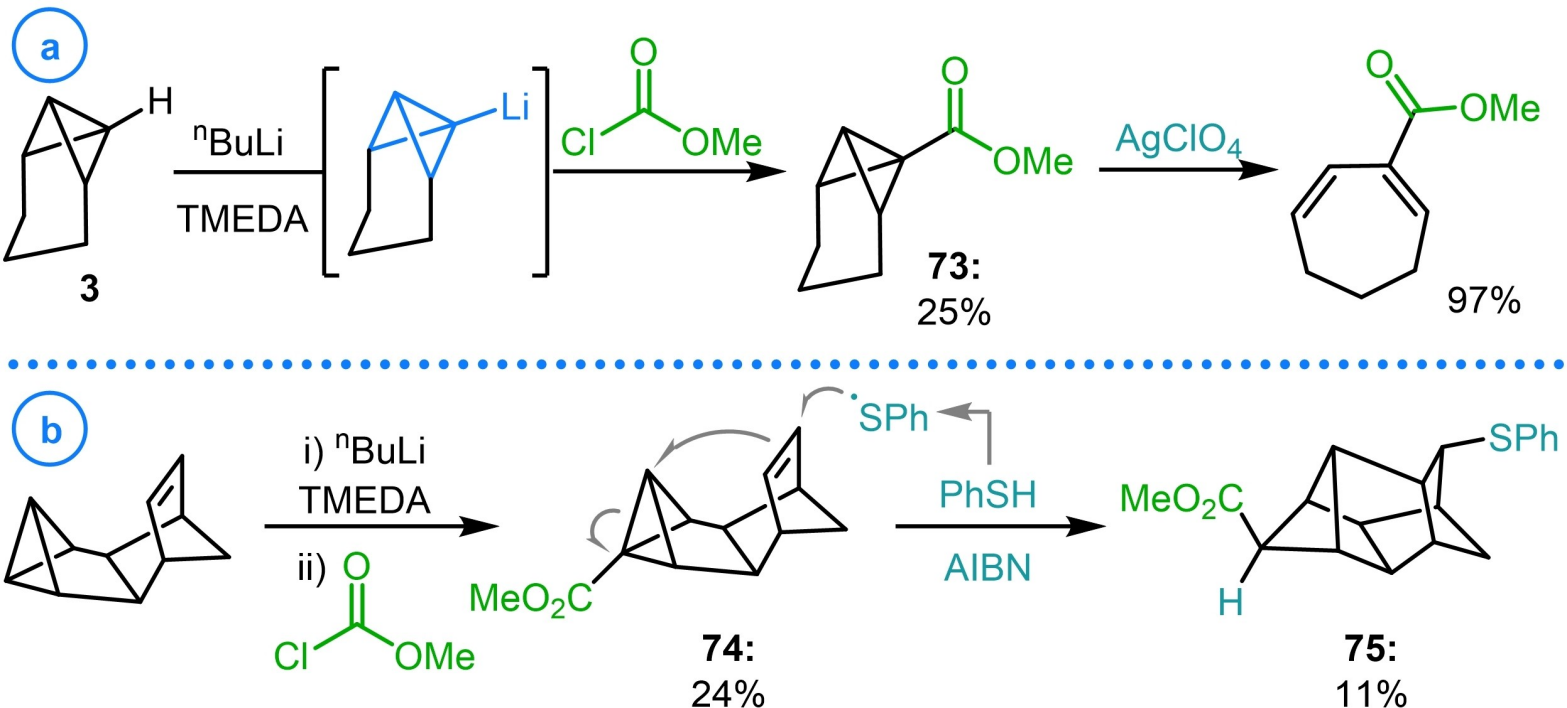
a) Paquette's study of the Ag(I)‐catalyzed rearrangement of electron deficient TCH‐based structure **73**. b) Christl's synthesis and intramolecular ring‐closure of electron deficient benzvalene Diels‐Alder adduct **74**.

The synthesis of bicyclo[1.1.0]butanes bearing electron withdrawing groups represents a field of considerable interest due to the ability of these strained species to act as Michael/Giese acceptors.[^19^, ^20^, ^21^, ^24^, ^25^, ^26^] In this regard, acylation of metalated bicyclo[1.1.0]butanes is a desirable method to access these compounds as it can be performed at a late stage to rapidly install a wide variety of ketone, ester or amide groups and, through deprotonation, can be achieved on an unfunctionalized bridgehead position. The first examples of this reactivity were achieved by employing chloroformates to install ester groups on bicyclo[1.1.0]butanes.[^40^, ^73^] In their previously mentioned 1974 study on the Ag(I)‐catalyzed rearrangement of tricyclo[4.1.0.0^2,7^]heptanes (see Scheme 2), Paquette and coworkers established that electron‐deficient bicyclo[1.1.0]butane **73** produced remarkably different product distributions compared to alkylated analogues (Scheme 18a).^[63]^ The synthesis of **73** was achieved through the bridgehead deprotonation of TCH (**3**) followed by inverse addition of the resulting organolithium to excess methyl chloroformate. In 1998, Christl and coworkers used this same synthetic procedure to generate electron deficient benzvalene Diels–Alder adduct **74** which was shown to react with thiyl radicals and subsequently undergo intramolecular cyclization onto the strained system to give scaffold **75** (Scheme 18b).^[111]^

**Scheme 19:**
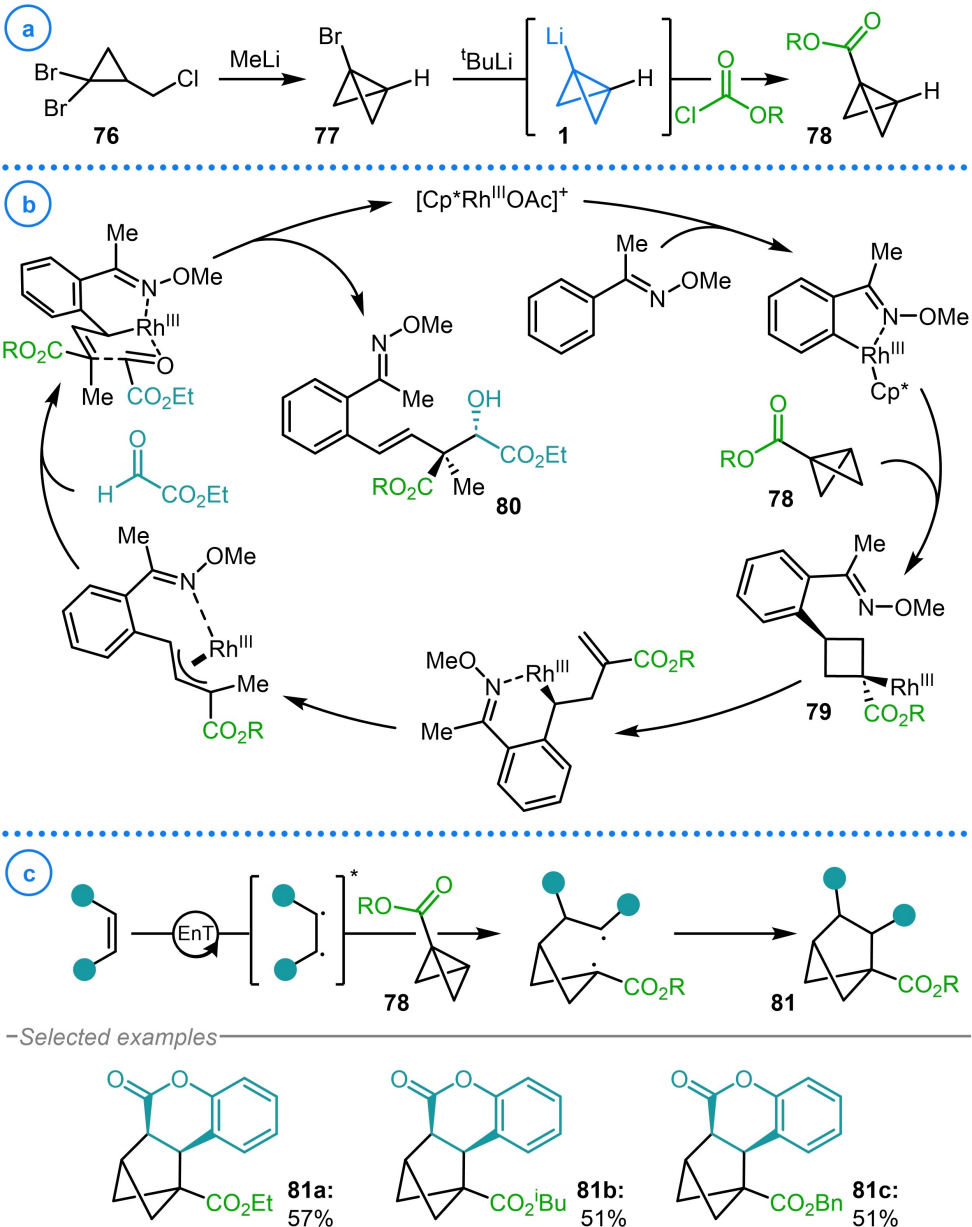
a) Glorius’ use of bicyclo[1.1.0]butyl lithium in the synthesis of **78**. b) Glorius’ strain‐release‐driven [2π+2σ]‐photocycloaddition reactions of electron deficient bicyclo[1.1.0]butanes with coumarins.

More recently this protocol has been exploited by Glorius and coworkers to generate bicyclo[1.1.0]butyl esters **78** (Scheme 19a). According to previous reports, bicyclo[1.1.0]butyl lithium (**1**) was synthesized from cyclopropane **76** and subsequently reacted with various chloroformates. The σ‐bond of these strained building blocks was shown to insert into Rh−C bonds to give Rh‐cyclobutyl intermediates (**79**) that were used for the diastereoselective construction of quaternary carbon‐containing ring‐opened products **80**.^[112]^ In a later publication, these same starting materials were demonstrated to undergo strain‐release‐driven [2π+2σ]‐photocycloaddition reactions with coumarins to access bicyclo[2.1.1]hexane structures (**81**) in a diastereoselective fashion (Scheme 19b).^[113]^ This was achieved by employing visible‐light‐mediated triplet sensitization to excite the heterocyclic olefin to a triplet state that undergoes regioselective addition to electron deficient bicyclo[1.1.0]butanes (**78**). Following this, intersystem crossing and radical recombination deliver the targeted bicyclo[2.1.1]hexane cycloadducts **81 a**–**81 c**. These studies elegantly display the potential of the highly strained σ‐bond of bicyclo[1.1.0]butane to undergo π‐like reactivity due to the increased p orbital character in this bridging bond.

**Scheme 20:**
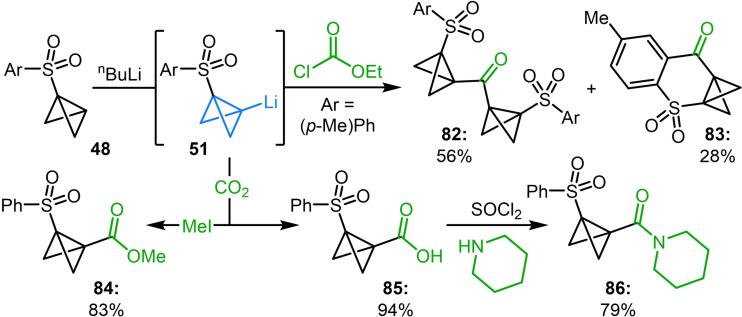
Gaoni's reaction of lithiated 1‐(arylsulfonyl)bicyclo[1.1.0]butane **51** with ethyl chloroformates and CO_2_.

Despite the aforementioned success of installing esters on bicyclic scaffolds, Gaoni and coworkers discovered that when a bicyclo[1.1.0]butane already containing an electron withdrawing sulfone group (**48**) was deprotonated, the reaction with ethyl chloroformate resulted in the exclusive formation of undesired side‐products **82** and **83** in 56 % and 28 % respectively (Scheme 20).^[99]^ These arise from double addition of the bicyclo[1.1.0]butyl lithium fragment to the chloroformate and from the *ortho* deprotonation and intramolecular cyclization of the desired product. The selectivity profile could be improved by carefully controlling the stoichiometry of base, although **82** was still observed to form in this reaction.[^48^, ^114^] However, this issue could be alleviated by reacting the bicyclo[1.1.0]butyl anion directly with CO_2_ before quenching with MeI to access the desired ester **84** free from side‐products. The intermediate carboxylate could also be used to obtain free carboxylic acid **85** which was subsequently transformed into the corresponding piperidine amide (**86**).^[48]^

**Scheme 21:**
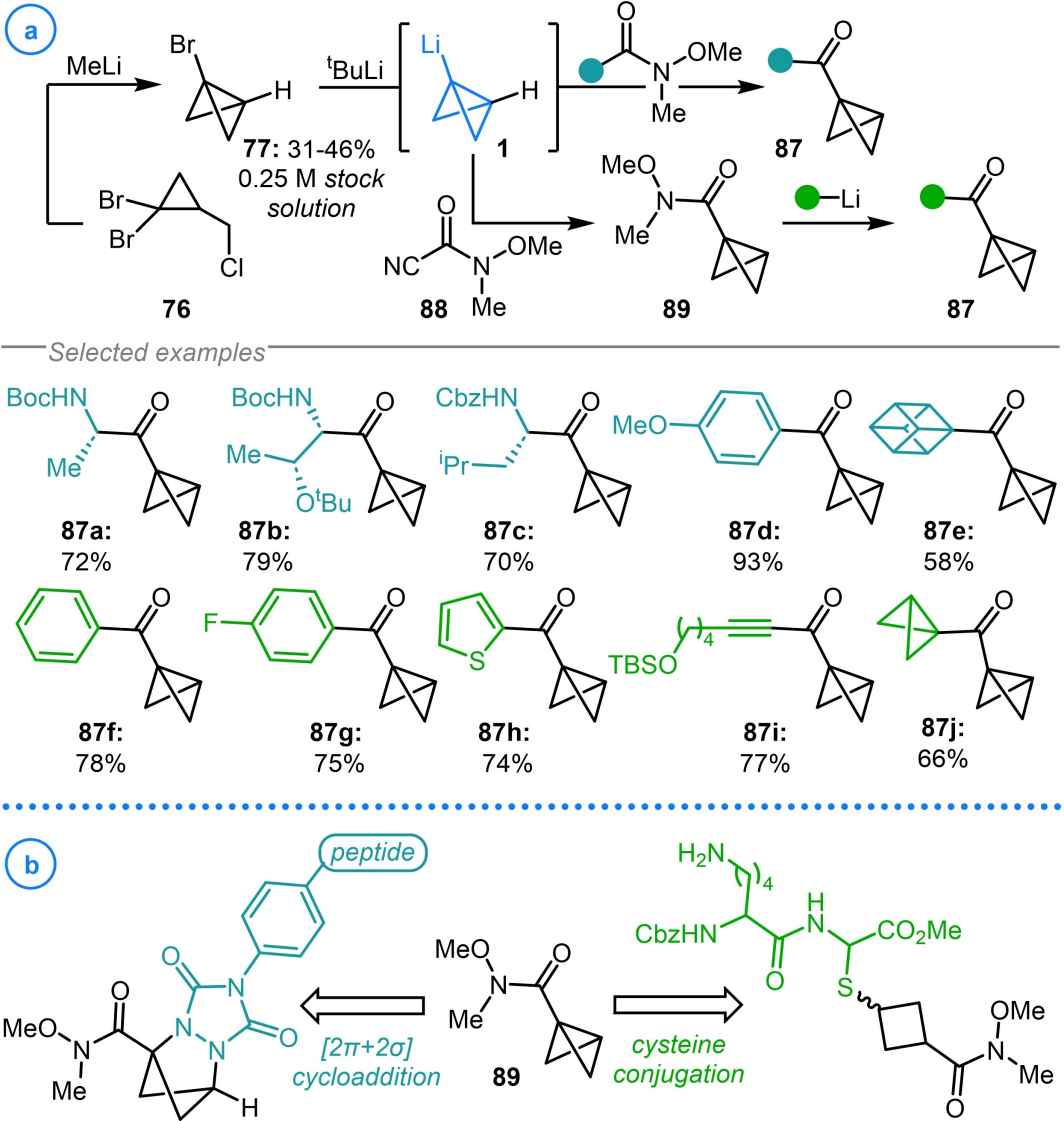
a) Malin's addition of bicyclo[1.1.0]butyl lithium to Weinreb amides. b) Application of bicyclo[1.1.0]butyl amide **89** in click‐type conjugation reactions.

Alternatively, further nucleophilic addition of organometallic intermediates to the products during the synthesis of acylated bicyclo[1.1.0]butanes can be avoided by employing Weinreb amide electrophiles. This strategy was explored by Malins and coworkers who demonstrated that a broad range of bicyclo[1.1.0]butyl ketones (**87 a**–**87 e**) could be accessed using this methodology, including those electrophiles derived from *N*‐protected amino acids (Scheme 21a).^[115]^ As well as this, bicyclo[1.1.0]butyl Weinreb amide **89** was synthesized from Manders‐type reagent **88** and was demonstrated to serve as a common intermediate for the addition of a variety of organometallics (**87 f**–**87 j**). Unique to this work was the use of a stock solution of 1‐bromo‐bicyclo[1.1.0]butane **77** rather than generating this species in situ and immediately subjecting it to lithium‐halogen exchange conditions to access bicyclo[1.1.0]butyl lithium **1**. The utility of the carbonylated bicyclo[1.1.0]butane products was demonstrated by their ability to undergo click‐type conjugation reactions with cysteine residues^[115]^ and, in a later publication, participate in [2π+2σ]‐cycloaddition reactions with peptide labelled triazolinediones (Scheme 21b).^[116]^

**Scheme 22:**
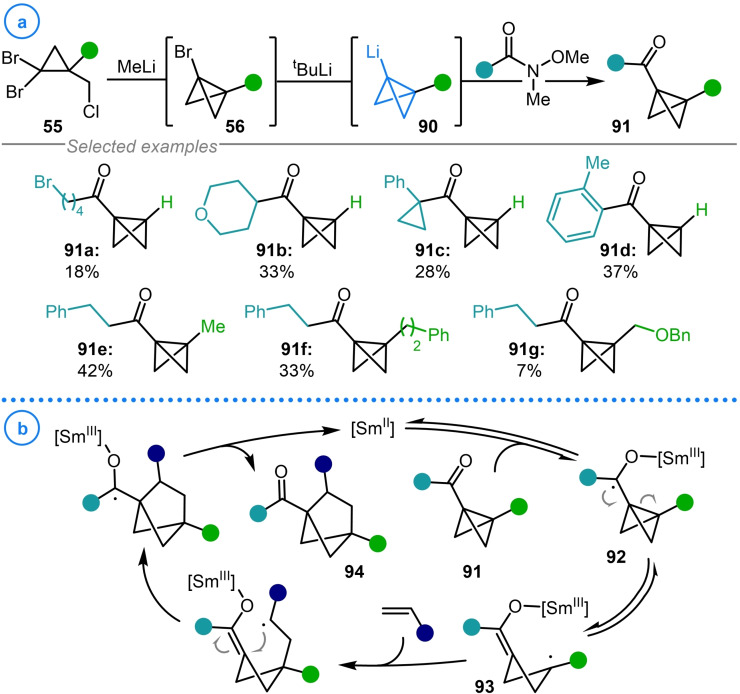
a) Bicyclo[1.1.0]butyl lithium as an intermediate in Procter's synthesis of bicyclo[1.1.0]butyl ketones. b) Mechanism of SmI_2_‐catalyzed formal [2π+2σ]‐cycloaddition reactions between bicyclo[1.1.0]butyl ketones and alkenes.

A similar synthetic strategy has also been recently exploited by Procter and coworkers to access bicyclo[1.1.0]butyl ketones (**91 a**–**91 g**) from bicyclo[1.1.0]butyl lithium **90** and Weinreb amides (Scheme 22a).^[117]^ Here, the resulting products were engaged in SmI_2_‐catalyzed formal [2π+2σ]‐cycloaddition reactions with a broad range of electron deficient olefins. The reactivity is dependent on the presence of the adjacent ketone which is reduced to ketyl radical **92** before fragmentation of the strained bicycle gives species **93** (Scheme 22b). Subsequent radical trapping with the alkene delivers a radical intermediate which then undergoes ring‐closure and back electron transfer to construct the bicyclo[2.1.1]hexyl scaffold (**94**). The necessity of the ketone functionality in the starting materials highlights the importance of developing synthetic methods to efficiently access a diverse array of these structures from readily available precursors.

**Scheme 23:**
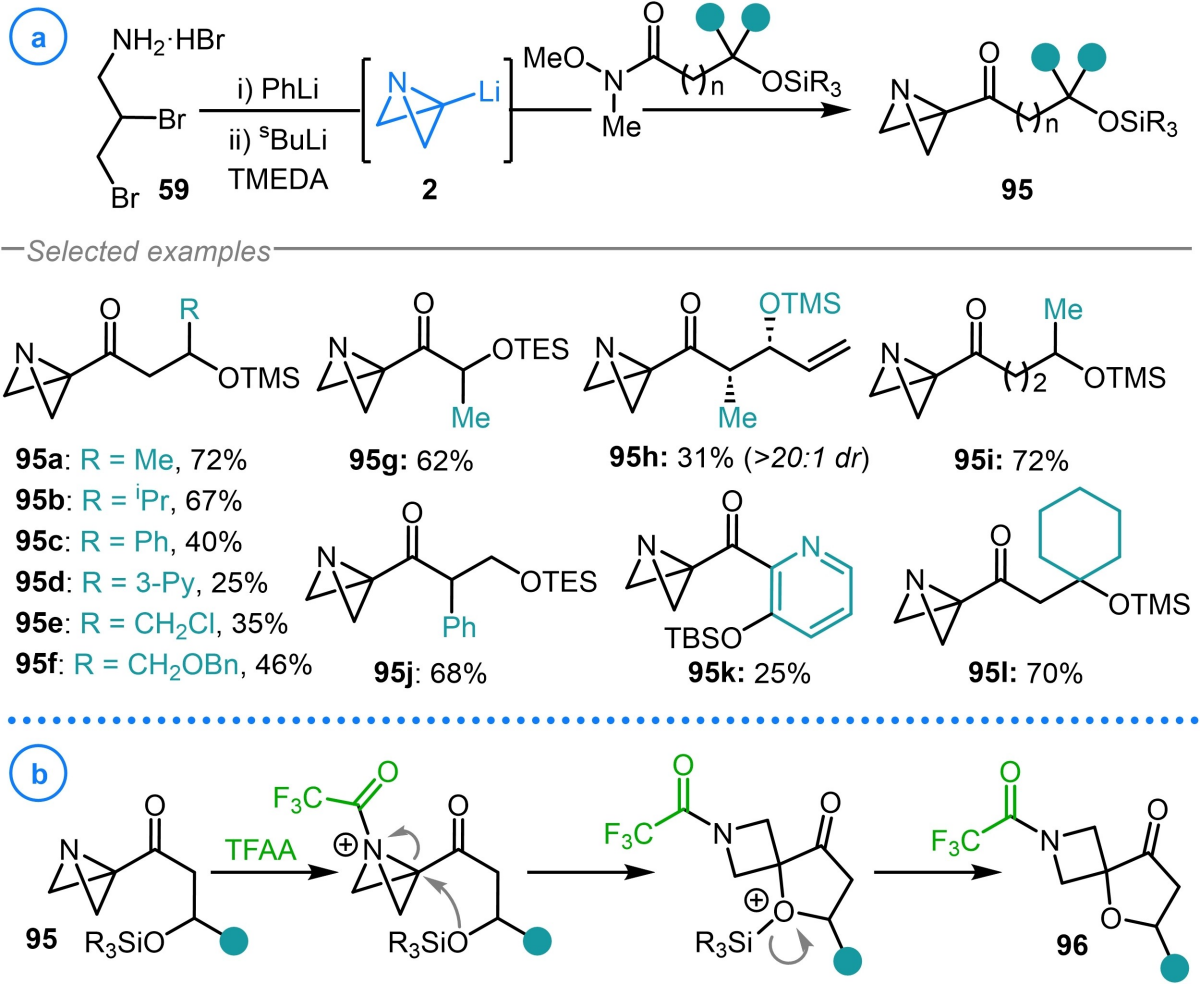
a) Azabicyclo[1.1.0]butyl lithium (**2**) as an intermediate in Aggarwal's synthesis of silyl ether tethered bicyclo[1.1.0]butyl ketones. b) Electrophile‐induced strain‐release‐driven spirocyclization reaction of **95**.

Analogously, Aggarwal and coworkers demonstrated that the coupling of azabicyclo[1.1.0]butyl lithium **2** with Weinreb amides allowed rapid access to azabicyclo[1.1.0]butyl ketones with tethered silyl ether groups **95 a**–**95 l** (Scheme 23a).^[118]^ This synthesis was performed in the context of using azabicyclo[1.1.0]butyl lithium as a carbenoid fragment to access spirocyclization precursors (**95**). Such species, upon acylation of the bicyclic nitrogen, could undergo intramolecular strain‐release‐driven ring‐closure reactions to deliver azetidine spirocycles **96** (Scheme 23b).

### 5.4 (Aza)Bicyclo[1.1.0]butyl boronate complexes

**Scheme 24:**
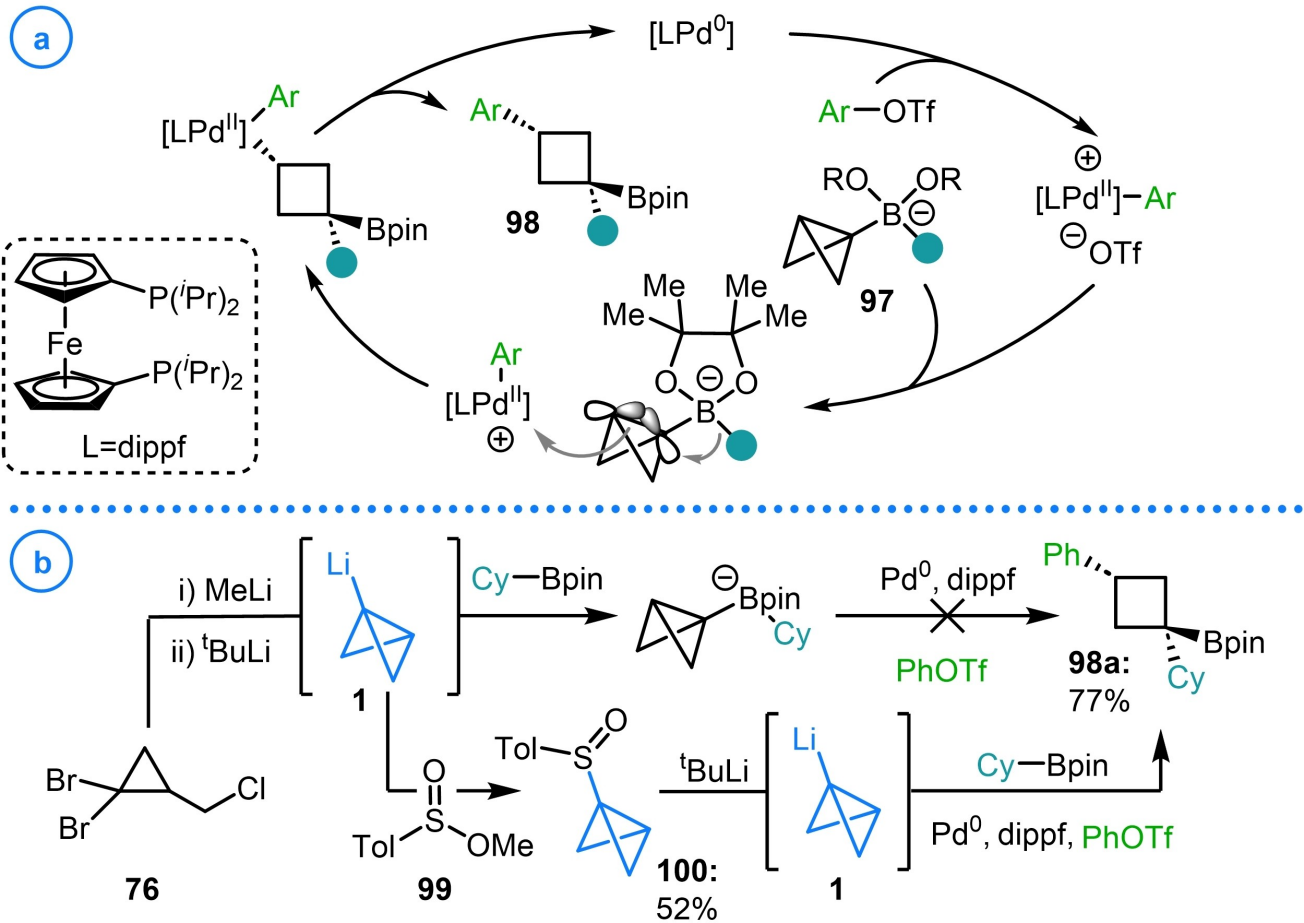
a) Mechanism of Aggarwal's σ‐bond carbopalladation reaction enabled by strained boronate complexes (**97**). b) Aggarwal's use of sulfoxide **100** for the halide‐free generation of bicyclo[1.1.0]butyl lithium.

The coupling of bicyclo[1.1.0]butyl lithium **1** with boronic esters to generate highly strained boronate complexes has been pioneered by Aggarwal and coworkers. The resulting electron rich bicyclo[1.1.0]butyl boronate complexes (**97**) have been shown to interact with electrophilic Pd(II) complexes, generated from the oxidative addition of a Pd(0) catalyst into an aryl triflate (Scheme 24a).^[55]^ This coordination induces a stereoselective strain‐release‐promoted 1,2‐metallate rearrangement of the carbon‐boron bond to open the bicycle and simultaneously form a new C−Pd bond. The high diastereoselectivity of this migration process was stated to arise from the antiperiplanar alignment of the central bond of the bicyclo[1.1.0]butane fragment and the migrating substituent. As a consequence, the Pd(II) complex approaches the bicycle from the concave face to avoid the steric clash with the pinacol ligand and to maximize overlap with the p orbitals of the bridging bond. Finally, reductive elimination regenerates the Pd(0) catalyst and delivers substituted cyclobutyl boronic ester products **98**.

Of relevance to the present review was the observation that no cross‐coupling product was formed upon the in situ generation of bicyclo[1.1.0]butyl lithium from dibromo cyclopropane **76** (Scheme 24b).^[55]^ It was hypothesized that the presence of halide salts could lower the electrophilicity of the Pd(II) complex by ligating to the vacant coordination site, necessitating the conception of a new method for the halide‐free generation of bicyclo[1.1.0]butyl lithium.^[119]^ This was achieved via the synthesis of bicyclo[1.1.0]butyl sulfoxide **100**, accessed from the reaction of bicyclo[1.1.0]butyl lithium with sulfinate ester **99**. This easily handled, bench‐stable precursor could be purified by column chromatography and stored indefinitely. The desired organolithium intermediate could then be unveiled on demand upon exposure of **100** to ^t^BuLi to facilitate lithium‐sulfoxide exchange.^[55]^ Another benefit of this approach is that the formation of bicyclo[1.1.0]butyl lithium can be achieved in the presence of the boronic ester which can rapidly sequester this highly reactive species which decreases the chance of deleterious side reactions occurring.

**Scheme 25:**
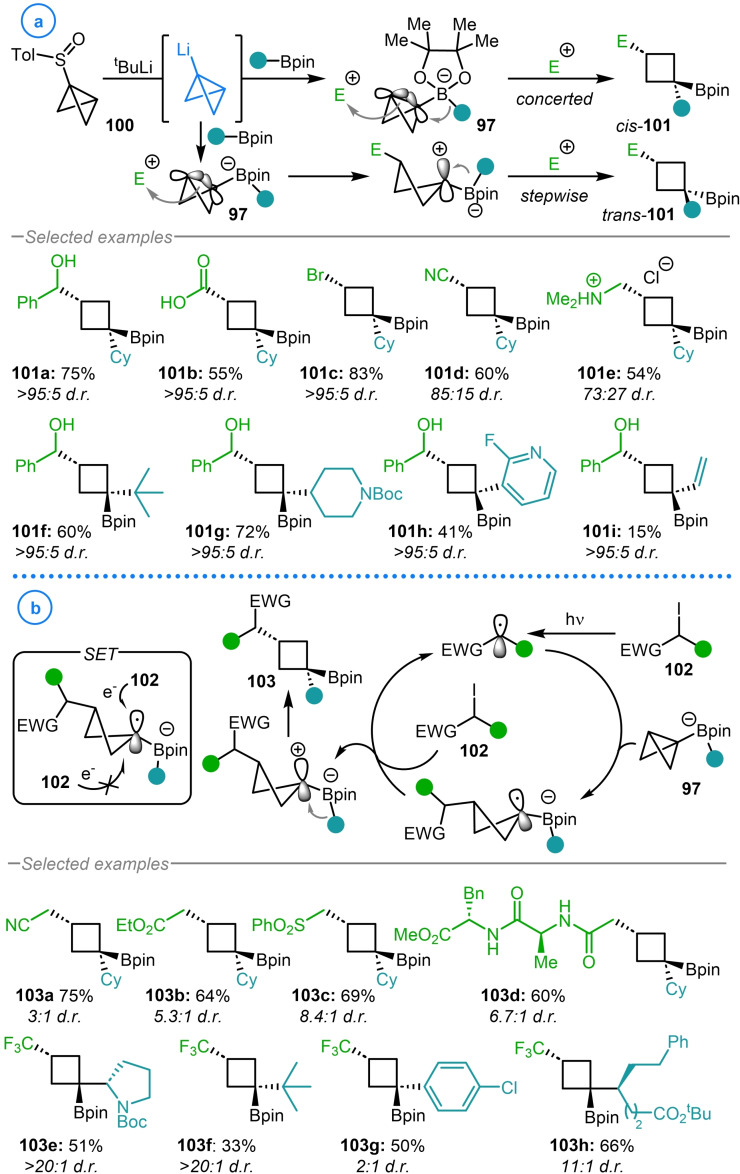
a) Possible concerted and stepwise pathways in Aggarwal's electrophilic activation of bicyclo[1.1.0]butyl boronate complexes (**97**). b) Aggarwal's addition of electron deficient radicals to bicyclo[1.1.0]butyl boronate complexes.

The generality of this approach was later established when Aggarwal and coworkers reported the activation of the same bicyclo[1.1.0]butyl boronate complexes (**97**) with a wide range of electrophiles (Scheme 25a).^[120]^ These included aldehydes, ketones, imines, acyl chlorides, chloroformates, carbon dioxide and even electrophilic sources of chlorine, bromine and iodide to provide alternative cyclobutyl boronic esters (**101 a**–**101 i**). Although this methodology also delivered the desired products with high selectivity for the ‘*cis*’ isomer, in the case of a handful of electrophiles the d.r. was slightly diminished by a competitive stepwise mechanism from which 1,2‐migration is non/less selective compared to the corresponding concerted pathway (Scheme 25a).

In a mechanistically distinct approach, it was shown that electron deficient carbon‐centered radicals derived from alkyl iodides (**102**) could also add to the β‐carbon of cyclobutyl boronate complexes **97** (Scheme 25b).^[121]^ Using this strategy, complementary cyclobutyl boronic ester products (**103 a**–**103 h**) could be accessed with good selectivity for the ‘*cis*’ diastereomer. Despite proceeding through a planar radical intermediate, selectivity was hypothesized to arise from the single electron‐transfer step in which the oxidant **102** approaches from the less hindered face of the radical and the subsequent 1,2‐migration is more facile than C−B bond rotation. However, subsequent spectroscopy studies have suggested the intermediacy of an α‐iodoboronate intermediate from which stereospecific 1,2‐migration can occur to give the cyclobutane product.^[122]^

**Scheme 26:**
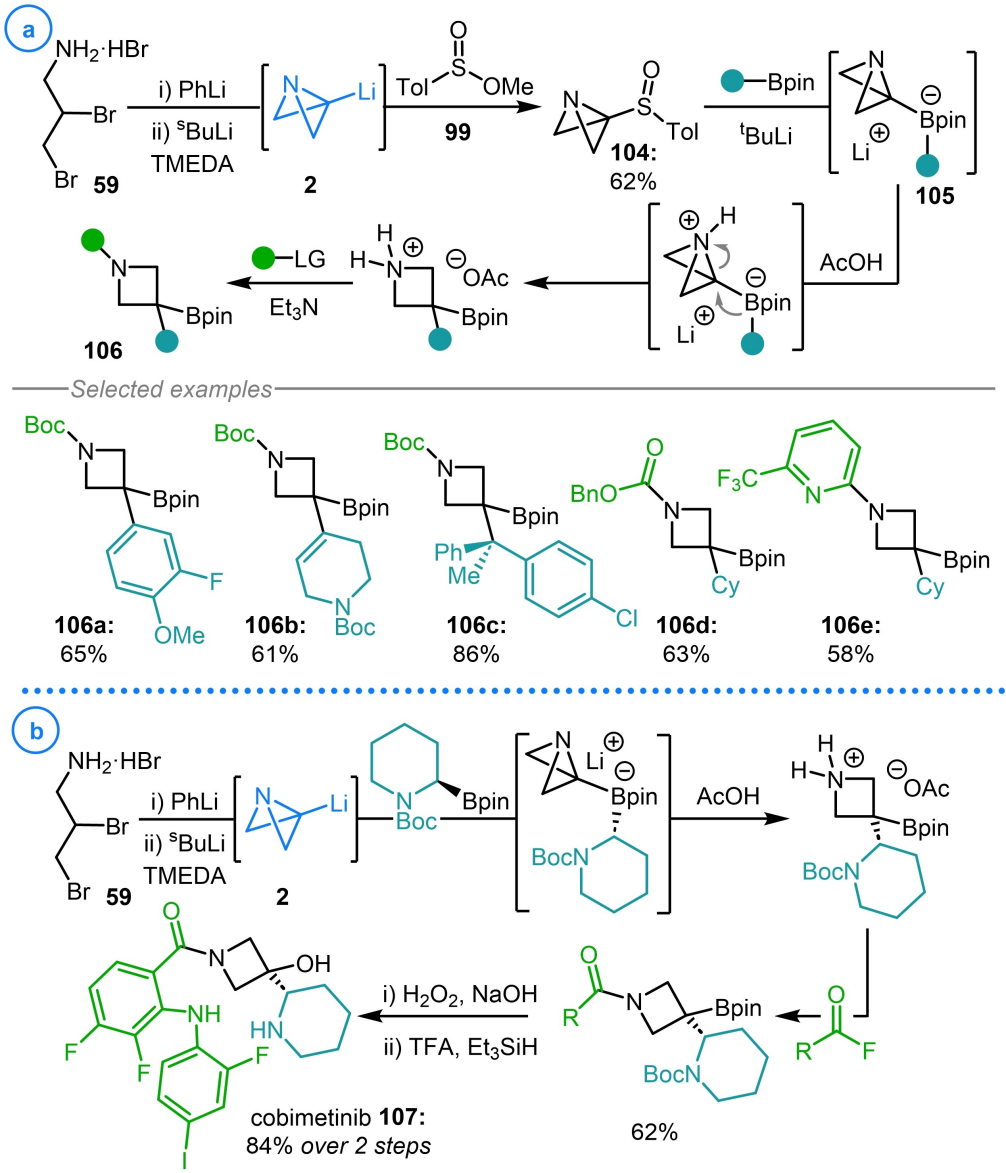
a) Aggarwal's strain‐release‐driven 1,2‐migration reaction of azabicyclo[1.1.0]butyl boronate complexes (**105**). b) Application to the synthesis of cobimetinib.

The synthesis of the analogous azabicyclo[1.1.0]butane sulfoxide **104**, from dibromoamine **59** and sulfinate ester **99**, led to the exploration of whether similar reactivity could be achieved to access azetidinyl boronic esters **106** (Scheme 26a).^[51]^ Accordingly, lithium‐sulfoxide exchange of **104** with ^t^BuLi in the presence of a boronic ester coupling partner led directly to azabicyclo[1.1.0]butyl boronate complexes **105** via azabicyclo[1.1.0]butyl lithium **2**. Activation of the N‐heterocycle with AcOH was demonstrated to trigger the desired 1,2‐migration and the resulting secondary amine intermediate could then be functionalized to aid purification. Due to the broad scope of both the boronic ester and the electrophile used to functionalize the resulting azetidine intermediate, this methodology represents a highly modular approach toward the synthesis of substituted azetidines (**106 a**–**106 e**). It must also be noted that without the restrictions of requiring halide‐free conditions, ABB−Li could also be accessed directly from dibromoamine **59** and was demonstrated as such in the synthesis of the MEK inhibitor cobimetinib (**107**, Scheme 26b).^[51]^

**Scheme 27:**
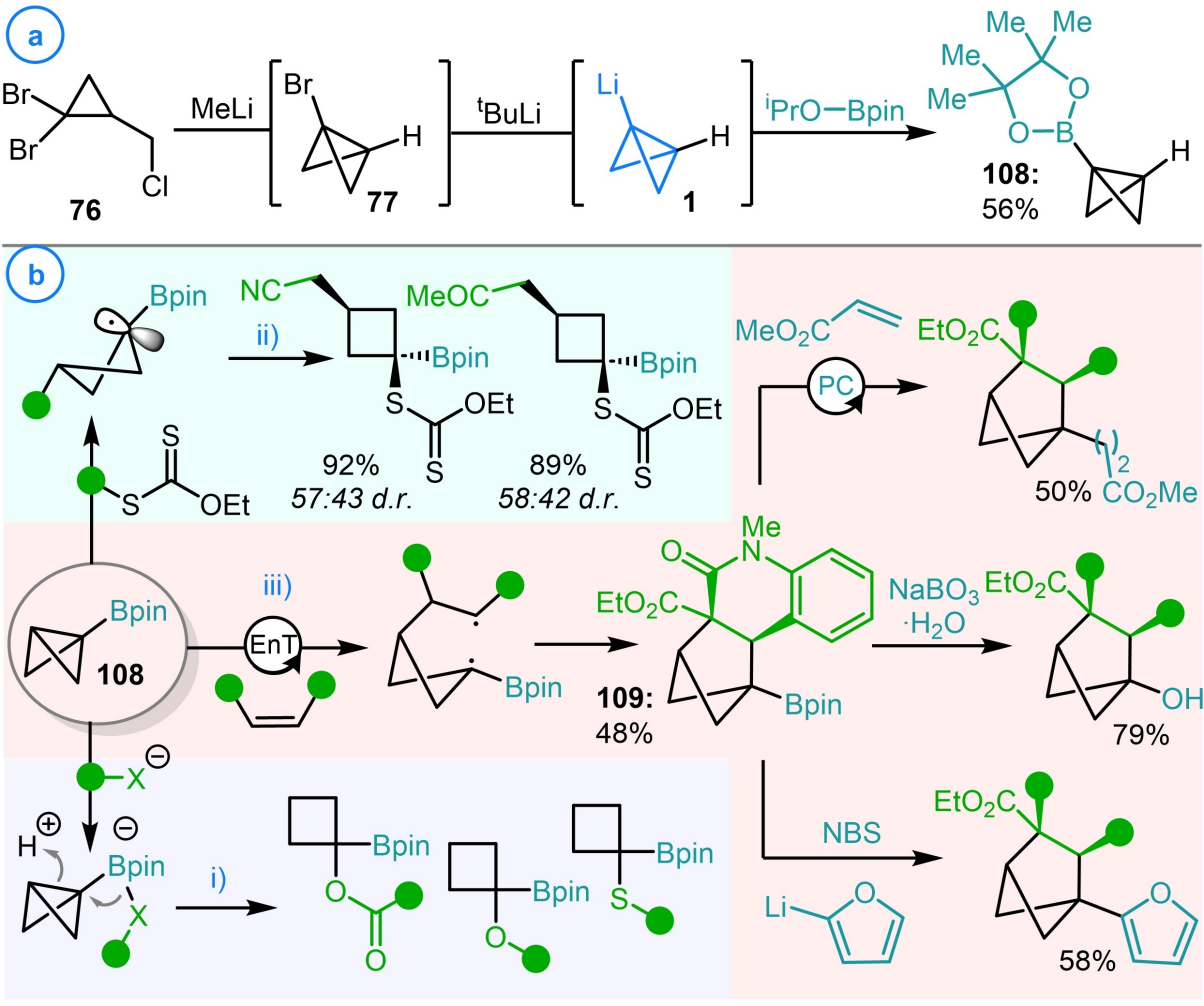
a) Aggarwal's synthesis of BCB‐Bpin (**108**). b) Synthetic applications of **108** as reported by Aggarwal, Zard and Glorius.

As well as coupling to boronic esters, Aggarwal and coworkers demonstrated that bicyclo[1.1.0]butyl lithium (**1**) could react with borate esters such as 2‐isopropoxy 4,4,5,5‐tetramethyl‐1,3,2‐dioxaborolane (^i^PrOBpin) to generate the isolable and highly versatile fragment BCB‐Bpin (**108**, Scheme 27a).^[123]^ This species was subsequently shown to generate boronate complexes with a range of heteroatom‐centered nucleophiles which directly underwent strain‐release‐driven 1,2‐metallate rearrangements upon protonation of the bicyclo[1.1.0]butane fragment (Scheme 27bi). An alternative application of BCB‐Bpin (**108**) was reported by Zard and coworkers who demonstrated that this fragment could undergo radical addition reactions with alkyl xanthantes (Scheme 27bii).^[124]^ The relative stability of the resulting α‐boryl cyclobutane radical facilitates this initial addition allowing it to act as a Giese‐type acceptor. This radical stability was also exploited by Glorius and coworkers who applied BCB‐Bpin as a substrate in their previously discussed (see Scheme 19) [2π+2σ]‐cycloaddition reactions (**109**, Scheme 27biii).^[113]^ The benefits of using a boron handle instead of an ester as a radical stabilizing group was clearly demonstrated by the number of subsequent transformations that the borylated bicyclo[2.1.1]hexane products could undergo. Currently, the synthesis of BCB‐Bpin (**108**) can only be achieved by utilizing bicyclo[1.1.0]butyl lithium as an intermediate, highlighting the unique synthetic opportunities that metalated bicyclo[1.1.0]butanes can offer.

### 5.5 Metal‐catalyzed cross‐coupling

**Scheme 28:**
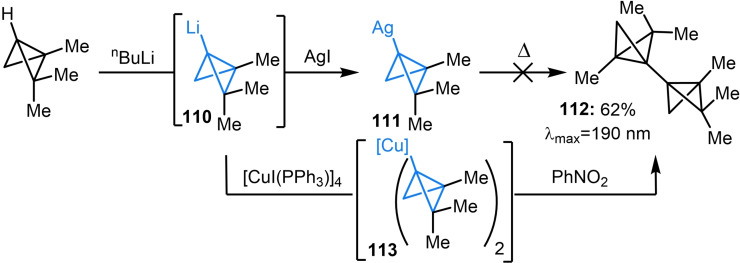
Moore and Costin synthesis of bicyclo[1.1.0]butane dimer **112**.

Although the majority of synthetic strategies discussed above have involved bicyclo[1.1.0]butyl lithium species, transmetalation to generate alternative organometallic intermediates has provided new avenues in carbon‐carbon bond construction. Accordingly, metalated bicyclo[1.1.0]butanes can participate in a range of cross‐coupling reactions in which metal insertion into the strained bridging bond is not observed as a competing pathway. In 1971, Moore and Costin endeavored to synthesize a dimer of bicyclo[1.1.0]butane to assess the extent of conjugation between the strained bonds of the bicycle.^[125]^ Addition of silver(I) iodide to organolithium **110** resulted in the formation of **111** which did not undergo the expected thermal homocoupling at elevated temperatures (Scheme 28).^[126]^ However, generation of the corresponding bicyclo[1.1.0]butyl Gilman reagent **113** could facilitate the homocoupling process upon oxidation with nitrobenzene. The UV absorption spectrum of dimer **112** showed a noticeable red shift compared to monomeric bicyclo[1.1.0]butanes, indicating that the strained central bonds can participate in conjugation. This same oxidative homocoupling procedure was later used by Szeimies and coworkers who further investigated the reactivity of tricyclo[4.1.0.0^2,7^]heptane dimers and trimers.^[127]^

**Scheme 29:**
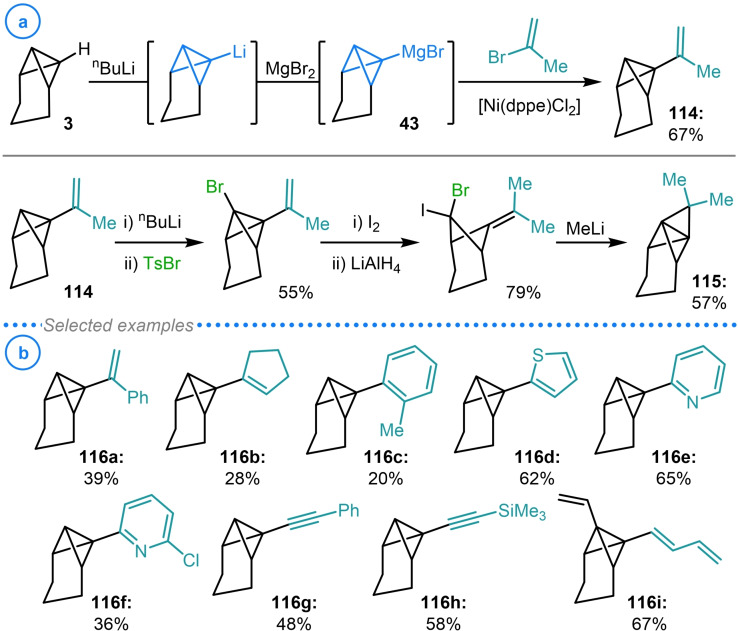
a) Szeimies’ Kumada coupling of TCH Grignard **43** with 2‐bromopropene and its application in the synthesis of [1.1.1]propellane **115**. b) Products generated from the cross‐coupling of **43** with alkenyl, alkynyl and (hetero)aryl halides.

The first example of bicyclo[1.1.0]butane cross‐coupling was reported by Szeimies and coworkers in 1988 and required the transmetalation of tricyclo[4.1.0.0^2,7^]heptyl lithium to the analogous Grignard reagent (**43**) with anhydrous MgBr_2_ (Scheme 29a).^[128]^ This intermediate was then engaged in Kumada‐type cross‐coupling with 2‐bromopropene in the presence of catalytic [Ni(dppe)Cl_2_] to generate **114** in 67 % yield. This species was used as an intermediate in the synthesis of [1.1.1]propellane **115**, achieved via bridgehead bromination, iodide addition, reduction and a final intramolecular carbene insertion reaction. The cross‐coupling procedure was also extended to other alkenyl and alkynyl halides as well as aryl and heteroaryl halides in later publications (**116 a**–**116 i**, Scheme 29b).[^129^, ^130^]

**Scheme 30:**
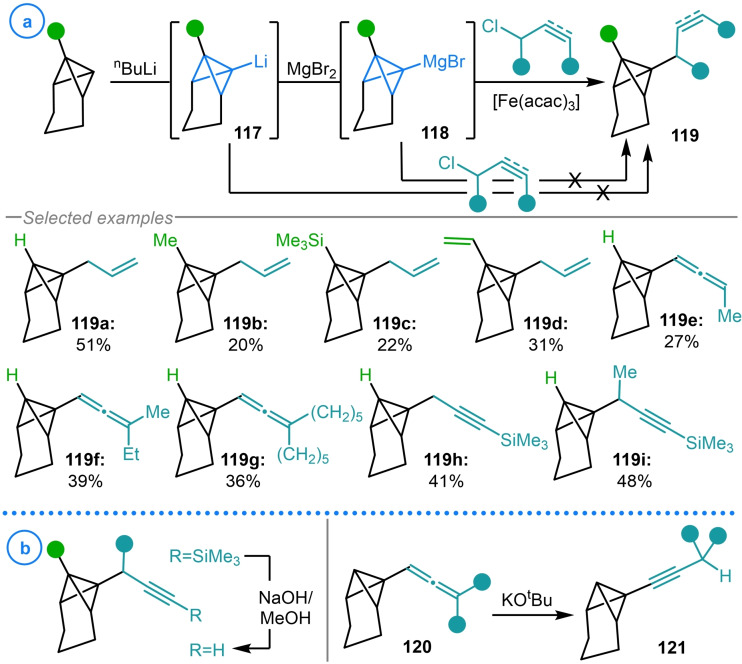
a) Szeimies’ iron‐catalyzed cross‐coupling of TCH Grignard **118** with vinyl and propargyl chlorides. b) Alkyne silyl deprotection and allene isomerization of cross‐coupling products.

The same group would eventually demonstrate that sp^3^/sp^3^ cross‐coupling can also be achieved by employing iron catalysis (Scheme 30a).^[131]^ Although it was shown that the desired carbon‐carbon bond forming reaction between vinyl/propargyl chlorides and tricyclo[4.1.0.0^2,7^]heptyl Grignards **118** could be achieved with Cu, Ni and Co complexes, Fe(acac)_3_ was found to deliver **119** in the highest yields. The necessity of the catalyst was also established when the direct reaction of either organolithium **117** or Grignard reagent **118** with the targeted electrophiles in the absence of a metal complex failed to deliver more than trace product. In the case of propargyl halides, competing S_N_2′ addition to form allenyl bicyclo[1.1.0]butanes (**119 e**–**119 g**) became preferential to cross‐coupling upon increasing the steric hinderance around the reactive propargylic site. To alleviate this, the terminal position of the alkyne was blocked with a trimethylsilyl group, allowing the exclusive formation of the propargylated product (**119 h**–**119 i**), with the silyl directing group easily removed in a subsequent step (Scheme 30b). Interestingly, propargyl bicyclo[1.1.0]butanes **120** were shown to undergo base‐catalyzed isomerization to the corresponding alkynyl compounds (**121**) without inducing ring‐opening of the strained bicycle.^[131]^

**Scheme 31:**
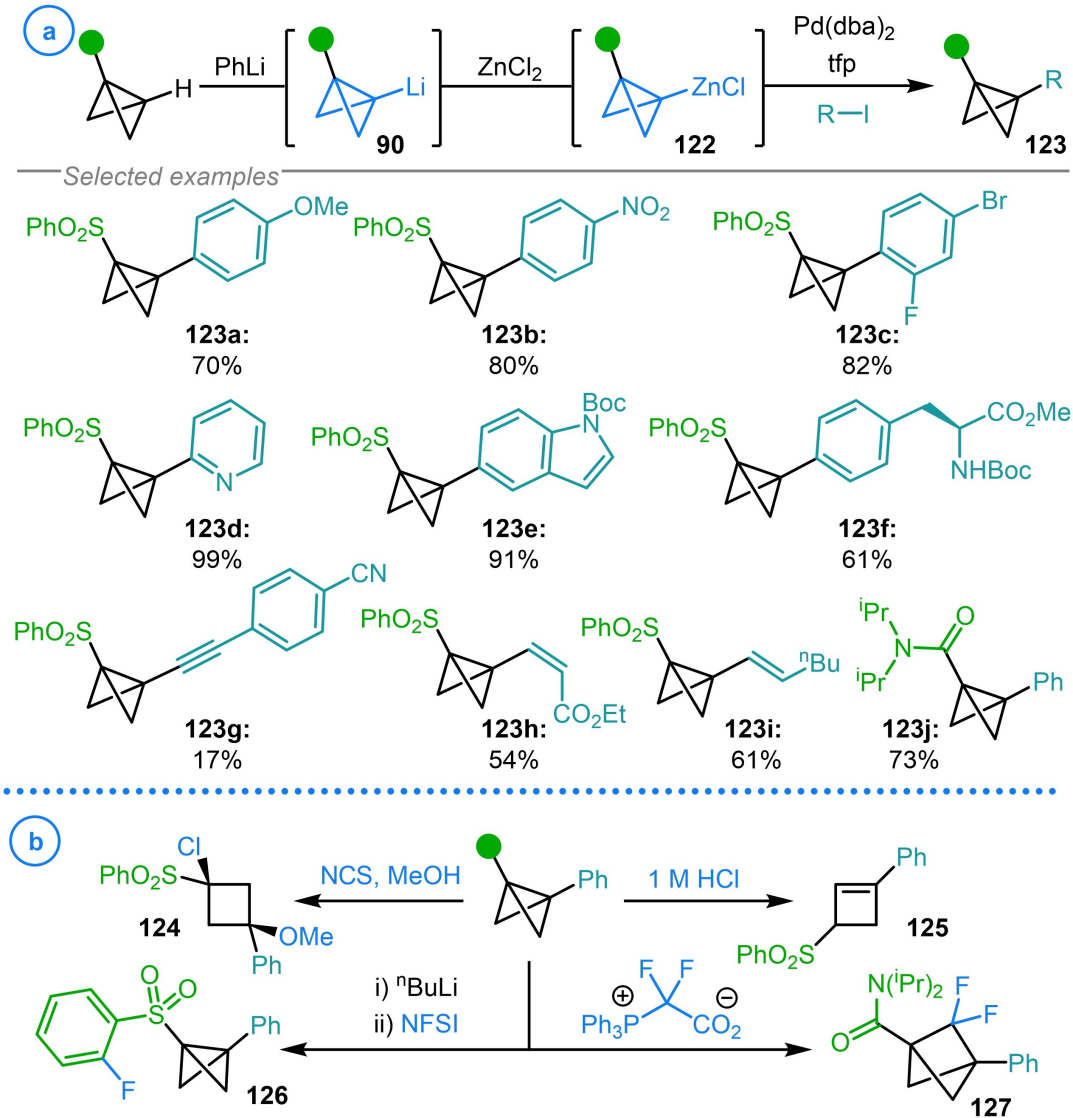
a) Anderson's Negishi cross‐coupling between bicyclo[1.1.0]butyl zinc species (**122**) and (hetero)aryl, alkenyl and alkynyl halides. b) Derivatization of arylated bicyclo[1.1.0]butane products.

In 2021, Anderson and coworkers greatly expanded the scope of potential coupling partners through the generation of organozinc complexes **122** via the addition of ZnCl_2_ to lithiated bicyclo[1.1.0]butanes **90** (Scheme 31a).^[49]^ These Zn‐BCBs were shown to engage in Pd(0) catalyzed Negishi‐type cross‐coupling reactions with a diverse series of alkenyl, alkynyl, aryl and heteroaryl iodides whilst also demonstrating impressive tolerance to a wide range of functional groups (**123 a**–**123 j**). Key to achieving the desired reactivity was suppressing deleterious bicyclo[1.1.0]butane rearrangements known to occur in the presence of electrophilic Pd(II) species.^[27]^ These side reactions were inhibited by reducing the π‐acidity of the intermediate palladium complexes by employing the electron‐rich trifurylphosphine (tfp) ligand and limiting the lifetime of Pd(II) intermediates by using organozinc complexes to enhance the rate of transmetalation. This methodology allowed access to a variety of novel disubstituted aryl‐bicyclo[1.1.0]butanes, with the synthetic utility of these species demonstrated by their transformation to cyclobutanes (**124**), cyclobutenes (**125**), sulfone functionalized bicyclo[1.1.0]butanes (**126**) and difluorobicyclo[1.1.1]pentanes (**127**, Scheme 31b).^[49]^

## 6 Bridging Methylene Metalation

The ability to directly functionalize the bridgehead C−H bond of a bicyclo[1.1.0]butane fragment, via the corresponding organometallic intermediate, has allowed huge advancements to be made in the exploration of the reactivity of these strained species. However, appending substituents to the bridging methylene units remains a synthetic challenge and typically requires the preinstallation of the required group before the assembly of the strained bicycle. Methods that have been used for the synthesis of bridge‐substituted bicyclobutanes include intramolecular nucleophilic substitution^[132]^ and cyclopropanation[^43^, ^44^] reactions, as well as the intermolecular biocatalytic double insertion of carbenes into terminal alkynes.^[45]^

**Scheme 32:**
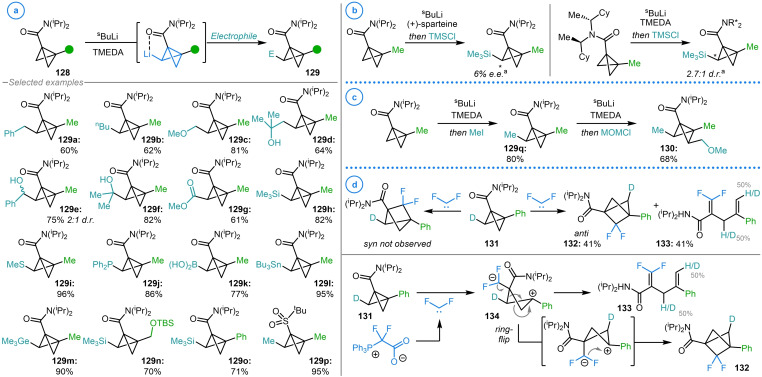
Anderson's study into the synthesis and reactivity of bridge lithiated bicyclo[1.1.0]butane. a) Bridge deprotonation and functionalization of bicyclo[1.1.0]butyl amide **128**. b) Attempted enantioselective deprotonation of bicyclo[1.1.0]butane bridging methylenes. ^a^ The identity of the major stereoisomer was not determined. c) Sequential bridge deprotonation and functionalization. d) Application of deuterated bicyclo[1.1.0]butane **131** in the mechanistic study of difluorocarbene insertion.

In 2021, Anderson and coworkers reported the first example of bicyclo[1.1.0]butane bridge metalation via the directed deprotonation of electron deficient bicyclo[1.1.0]butanes **128** with ^s^BuLi ligated with TMEDA (Scheme 32a).^[133]^ The resulting organolithium species was demonstrated to react with alkyl halides, epoxides, aldehydes, ketones, carbonyl chlorides and a broad range of heteroatom‐based electrophiles to install C−Si, C−S, C−P, C−B, C−Sn and C−Ge bonds on the bicyclo[1.1.0]butane framework (**129 a**–**129 p**). It is important to note that this deprotonation occurs with complete stereoselectivity for the *exo* C−H bond to deliver a single diastereomer of the products. This is predicted to be the site of kinetic deprotonation due to the directing ability of the bridgehead substituent, as well as the *exo* C−H bond being more sterically accessible than the corresponding *endo* position. This site may also be thermodynamically favored as calculations on the strain energies of various substituted bicyclo[1.1.0]butanes by Dill and coworkers showed that *endo* lithiated bicyclo[1.1.0]butane had a calculated strain energy of 5.0 kcal mol^−1^ higher than the analogous *exo* isomer.^[10]^ Furthermore, Wiberg showed that the *exo* hydrogen atom of bicyclo[1.1.0]butane displays a larger ^13^C−^1^H coupling constant than that for the *endo*, indicating a greater degree of s‐character to this bond and hence making it the more acidic site.^[1]^

Interestingly, Anderson and coworkers showed that some asymmetric induction could be achieved by employing either chiral lithium ligands or enantioenriched amides for the enantioselective functionalization of bicyclo[1.1.0]butanes (Scheme 32b).^[133]^ It was also demonstrated that trisubstituted bicyclo[1.1.0]butane (**129 q**) could undergo a further iteration of deprotonation and alkylation to deliver bicyclo[1.1.0]butanes in which all carbon atoms in the bicyclic framework have been selectively substituted (**130**, Scheme 32c). Therefore, a tetrasubstituted bicyclo[1.1.0]butane could theoretically be constructed from unsubstituted bicyclo[1.1.0]butane via the sequential metalation and functionalization of all positions on the strained scaffold. As well as utilizing the resulting products in a series of derivatizing ring‐opening reactions, Anderson and coworkers synthesized deuterium labelled **131** and used this compound to probe the mechanism and stereoselectivity of difluorocarbene insertion into the strained central bond of bicyclo[1.1.0]butane (Scheme 32d).^[30]^ Analysis of the stereochemistry in the difluorobicyclo[1.1.1]pentane product showed that the carbene added to the “bottom” face of the bicycle to give *anti*‐**132**. Additionally, the deuterium labelling pattern of diene side‐product **133** suggested the presence of a zwitterionic intermediate (**134**) in which fragmentation to **133** is competitive with ring flipping and subsequent ring‐closure. As discussed in Section 3, the selective isotopic labelling of bicyclo[1.1.0]butanes, made possible by the formation of metalated intermediates, has led to a greater understanding of the atypical mechanisms that strained fragments undergo.

## 7 Conclusions and Outlook

The use of metalated (aza)bicyclo[1.1.0]butanes in synthesis is currently experiencing a renaissance, as evidenced by the numerous reports in the last 5 years that have relied on such intermediates to undergo unique transformations or generate novel fragments. Since their discovery, these species have been demonstrated to participate in wide range of reactions with carbon and heteroatom electrophiles, as well as metal complexes, to facilitate the rapid diversification of (aza)bicyclo[1.1.0]butane‐containing compounds. Key to this is the relative acidity of the bridgehead C−H bonds which promotes the facile deprotonation and subsequent functionalization of an unsubstituted position on the carbon framework via the intermediacy of a metalated bicyclo[1.1.0]butane. Additionally, the late‐stage incorporation of deuterium atoms in strained fragments has led to the elucidation of numerous reaction mechanisms that involve strained bicycles.

Despite these great advancements there are still many areas within this field that have not been fully explored. These include the use of flow chemistry for the synthesis and subsequent reactivity of bicyclo[1.1.0]butane and the use of bicyclo[2.1.0]pentyl metalated species in synthesis. In comparison to bicyclo[1.1.0]butane, the chemistry of metalated nitrogen‐containing analogues have received less attention, despite functionalized azabicyclo[1.1.0]butanes displaying the potential to provide access to novel azetidine‐containing scaffolds upon bicyclic ring‐opening. The scope of electrophiles that metalated azabicyclo[1.1.0]butanes can react with has not been extensively examined and there are no examples to date of these species participating in cross‐coupling. Other questions that also remain unanswered in this field include whether the bridging methylene C−H bonds of azabicyclo[1.1.0]butane can be deprotonated and whether azabicyclo[1.1.0]butyl boronate species can participate in carbometalation reactions as has been shown for bicyclo[1.1.0]butane. The continued investigation into the inimitable reactivity of metalated bicycles will cement their importance within the field of organometallic chemistry.

## Conflict of interest

The authors declare no conflict of interest.

8

## Biographical Information

*Jasper L. Tyler received his MChem from The University of Sheffield in 2018. He is currently pursuing his PhD at the University of Bristol under the supervision of Professor Varinder K. Aggarwal. His research focuses on the development of novel synthetic strategies towards functionalized azetidines that utilize azabicyclo[1.1.0]butyl lithium*.

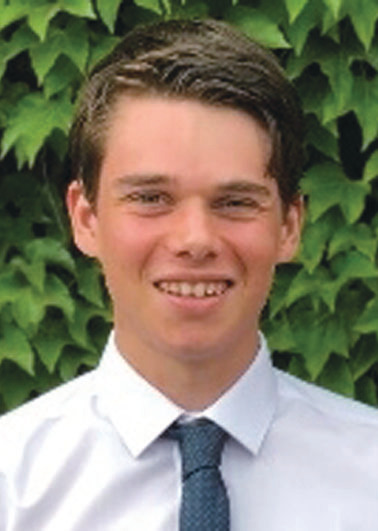

## Biographical Information

*Varinder K. Aggarwal studied chemistry at Cambridge University and received his Ph.D. in 1986 under the guidance of Dr. Stuart Warren. After postdoctoral studies (1986‐1988) under Prof. Gilbert Stork, Columbia University, he returned to the UK as a Lecturer at Bath University. In 1991 he moved to Sheffield University, where he was promoted to Professor in 1997. In 2000 he moved to Bristol University where he holds the Alfred Capper Pass Chair in Chemistry. His research interests include asymmetric synthesis, the development of chiral carbenoids and their use and subsequent applications in catalysis and synthesis*.

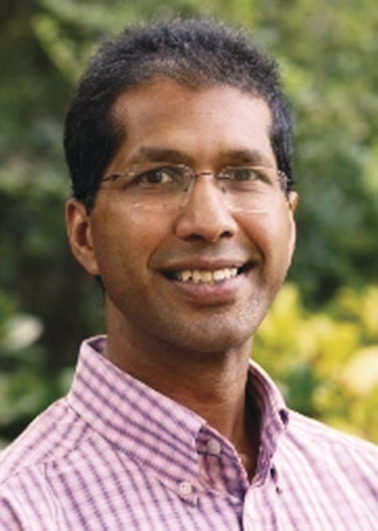

## Data Availability

Data sharing is not applicable to this article as no new data were created or analyzed in this study.
